# Highly diversified fungi are associated with the achlorophyllous orchid *Gastrodia flavilabella*

**DOI:** 10.1186/s12864-015-1422-7

**Published:** 2015-03-14

**Authors:** Tsunglin Liu, Ching-Min Li, Yue-Lun Han, Tzen-Yuh Chiang, Yu-Chung Chiang, Huang-Mo Sung

**Affiliations:** Institute of Bioinformatics and Biosignal Transduction, National Cheng Kung University, Tainan, Taiwan; Department of Life Sciences, National Cheng Kung University, Tainan, Taiwan; Department of Biological Sciences, National Sun Yat-Sen University, Kaohsiung, Taiwan

**Keywords:** Orchid, Mycorrhizal fungi, *Gastrodia flavilabella*, *Mycena* species, Mycoheterotrophic, Next-generation sequencing

## Abstract

**Background:**

Mycoheterotrophic orchids are achlorophyllous plants that obtain carbon and nutrients from their mycorrhizal fungi. They often show strong preferential association with certain fungi and may obtain nutrients from surrounding photosynthetic plants through ectomycorrhizal fungi. *Gastrodia* is a large genus of mycoheterotrophic orchids in Asia, but *Gastrodia* species’ association with fungi has not been well studied. We asked two questions: (1) whether certain fungi were preferentially associated with *G. flavilabella*, which is an orchid in Taiwan and (2) whether fungal associations of *G. flavilabella* were affected by the composition of fungi in the environment.

**Results:**

Using next-generation sequencing, we studied the fungal communities in the tubers of *Gastrodia flavilabella* and the surrounding soil. We found (1) highly diversified fungi in the *G. flavilabella* tubers, (2) that *Mycena* species were the predominant fungi in the tubers but minor in the surrounding soil, and (3) the fungal communities in the *G. flavilabella* tubers were clearly distinct from those in the surrounding soil. We also found that the fungal composition in soil can change quickly with distance.

**Conclusions:**

*G. flavilabella* was associated with many more fungi than previously thought. Among the fungi in the tuber of *G. flavilabella*, *Mycena* species were predominant, different from the previous finding that adult *G. elata* depends on *Armillaria* species for nutritional supply. Moreover, the preferential fungus association of *G. flavilabella* was not significantly influenced by the composition of fungi in the environment.

**Electronic supplementary material:**

The online version of this article (doi:10.1186/s12864-015-1422-7) contains supplementary material, which is available to authorized users.

## Background

Mycorrhizal association between plants and fungi is a common phenomenon in plants. In fact, most orchid species depend on interactions with mycorrhizal fungi for completing their life cycle, particularly during their early developmental stages because orchids lack endosperm or seed-based nutrient reserves [1]. Some orchid species are achlorophyllous through their entire life and must obtain carbon and nutrients for their growth and survival through mycorrhizal fungi [2]. It has been suggested that fully mycoheterotrophic orchids often show high specificity toward their mycorrhizal fungi [3-5]. Conversely, mycorrhizal association may vary in photosynthetic orchids [6,7]. Thus, identifying mycorrhizal fungi that are important for the survival and growth of mycoheterotrophic orchids may provide insights into the evolutionary dynamics between orchids and their fungal associates.

*Gastrodia* species form one of the largest achlorophyllous and mycoheterotrophic orchid genera, and are distributed throughout Oceania to Asia, including Australia, Vietnam, China, Taiwan, Japan and South Korea [8]. The rhizomes of *Gastrodia elata* are a prominent herbal medicine for human diseases such as vertigo, blackout and headache [9]. It has been suggested that *G. elata* depends on *Mycena* fungi for germination [10-12] and relies on *Armillaria* fungi for carbon and nutritional supply after germination [13,14]. In addition, some *Gastrodia* species are associated with litter- or wood-decaying fungi [4,15,16]. Ogura-Tsujita et al. first demonstrated that the adult *G. confusa* gain carbon through several wood- or litter-decaying *Mycena* species [4]. In contrast, *G. similis* was found to be associated largely with the saprotrophic *Resinicium* fungi [16]. Moreover, *G. sesamoides,* a common obligate mycoheterotrophic orchid species, relies on the saprotrophic fungi *Campanella* and *Marasmius* for its carbon supply instead of on an ectomycorrhizal fungal partner of a photosynthetic plant [15]. The above four *Gastrodia* species all depend on saprotrophic fungi for carbon and nutrition regardless of their geographic regions. In this study, we examined the fungal composition in the tubers of *G. flavilabella*, an endemic species of Taiwan [8], to identify the predominant fungi species associated with this plant. The identification is important because *Gastrodia* species do not always associate with the same fungal species for their growth.

Concurrent association of several fungi with an individual orchid is common, especially in photosynthetic orchids [17-20], suggesting the importance of studying the whole community of mycorrhizal fungi rather than the presence of individual fungal species. However, current mycorrhizal fungi identification requires microscopic identification and culturing or DNA segment cloning/sequencing, which may not provide sufficient resolution for the whole fungal community in the plant [14,16,17]. In this study, we used a deep sequencing approach to investigate the fungal community structure in the tubers of *G. flavilabella* and in the surrounding soil. We also investigated if *G. flavilabella* is preferentially associated with certain fungi.

Environmental and ecological factors may affect the extent of mycorrhizal preference of plant and the degree of dependence of plant on fungi for carbon and nutrients [7,21]. For example, the habitat characteristics and the presence of mycorrhizal fungi are important factors for the distribution of the orchid *Cypripedium californicum* because *C. californicum* is associated with multiple fungal families and may switch among different mycorrhizal fungi relatively easily [7]. In addition, McCormick et al. showed that the distribution of a mycoheterotrophic orchid, *Corallorhiza odontorhiza*, and three green orchids correlated strongly with the abundance of the required mycorrhizal fungi [22]. Thus, we also asked if the preferential fungal association of *G. flavilabella* is influenced by the fungal composition of the environment and/or by other environmental factors.

## Results

### Fungal samples and the 28S rDNA sequences

**Figure 1:**
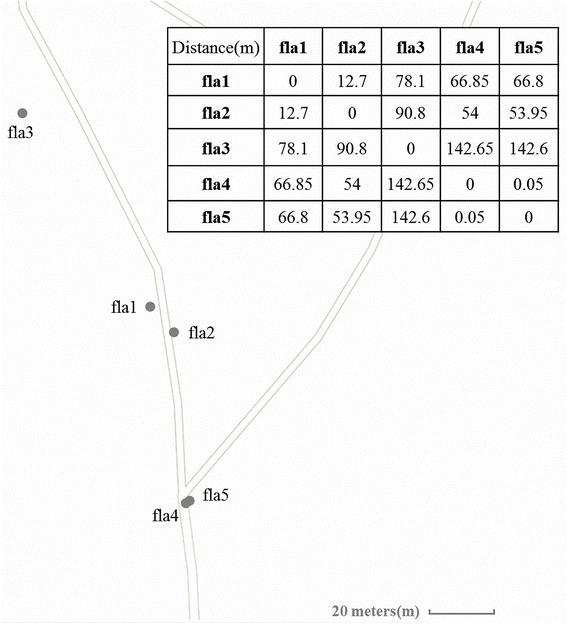
**Locations of the five Fla samples in the Hsitou area of Taiwan and the distances between them.**

To study the fungal communities in *G. flavilabella*, we collected five tuber samples (Fla1-5) and five soil samples from their surrounding soil (Methods) (Figure 1). From the genomic DNAs of each sample, the 28S rDNA segments were amplified and the PCR products were subjected to Illumina paired-end (PE) sequencing (Methods). There were at least 600,000 PE reads for each of the ten tuber and soil samples (Table 1). After merging PE reads and selecting high-quality merged reads (Methods), about 300,000 or more reads were obtained for analyzing each sample. The merged PE reads were deposited in NCBI SRA database under the SRA ID SRP054374. For all samples expect the Fla2 samples, ~60% of the data were of high-quality (Table 1). The lower percentages of high-quality merged reads in the Fla2 tuber and soil samples could be attributed to the shorter read length and lower sequencing quality. The length of the Fla2 paired reads (100 bp each) only allowed merged reads of size up to 190 bp, while the merged reads of other Fla samples could go up to 260 bp. In terms of mean quality, the Fla2 tuber and soil samples were the lowest at the tails of both paired reads (Additional file 1: Figure S1). Note that our soil samples contained higher fractions of long amplicons (>190 bp) than the tuber samples (Additional file 1: Figure S2). Because the Fla2 data did not allow long merged reads, we expected a lower percentage of merged reads in the Fla2 soil data than in the Fla2 tuber data; this was indeed the case (Table 1).

**Table 1:** **Information of the** ***Gastrodia flavilabella*** **(Fla) tuber and soil samples used in this study.**

***Gastrodia*** **sample**	**No. of 28S raw PE reads in tuber; soil**	**Length of read1; 2 (bp)**	**No. and % of high-quality merged reads in tuber; soil**
Fla1	928265; 943368	145; 125	567041 (61.1%); 598310 (63.4%)
Fla2	1223700; 1085260	100; 100	494672 (40.4%); 294985 (27.2%)
Fla3	716098; 776960	145; 125	432956 (60.5%); 473080 (60.9%)
Fla4	613629; 626538	145; 125	359376 (58.6%); 416882 (66.5%)
Fla5	712647; 787557	145; 125	412503 (57.9%); 487634 (61.9%)

### Taxonomic classification of the amplicon data

We used a BLAST-based nearest neighbor approach for taxonomic classification of the merged reads, i.e., amplicon sequences (Methods). To prepare a reference database, 268614 LSU sequences were collected from NCBI (Methods), of which 120617, 95458 and 14540 were fungi, metazoan, and viridiplantae, respectively. Based on the alignment results, our fungal specific LSU primers not only captured the 28S rDNAs of fungi, but also those of viridiplantae and metazoan (Table 2). As expected, the percentages (33.9-66.0%) of viridiplantae sequences in the tuber samples were higher than those (1.2-4.0%) in the soil samples (Table 2). Most of the viridiplantae sequences in the Fla tuber samples were classified into the same family of Orchidaceae (Additional file 1: Figure S4a), and they likely corresponded to the *G. flavilabella* sequences (see Discussion). In all the soil samples except Fla2, the percentages of metazoan sequences were higher than those in the tuber samples. In the tuber samples, the non-zero percentages of metazoan sequences suggest the presence of metazoa in the tubers and/or contamination. However, even if contamination occurred, the extent was relatively small and should not alter our major findings (see Discussion). Note that some of the LSU references were not classified at all the seven taxonomic levels, which explained most of the unclassified sequences. The high percentage (32.9-56.5%, Table 2) of unclassified reads suggested a large fraction of novel species in the soil samples. In the following, we focused only on the fungal reads.

**Table 2:** **Statistics of the tuber and soil PE read data from different taxonomy domains**

***Gastrodia*** **sample**	**Fungi**	**Viridiplantae**	**Metazoa**	**unclassified**
	**read number (%)**	**read number (%)**	**read number (%)**	**read number (%)**
Fla1 tuber	285092 (50.3%)	192331 (33.9%)	49633 (8.8%)	39985 (7.1%)
Fla2 tuber	274684 (55.5%)	210637 (42.6%)	199 (0.0%)	9152 (1.9%)
Fla3 tuber	105434 (24.4%)	285955 (66.0%)	21696 (5.0%)	19871 (4.6%)
Fla4 tuber	173314 (48.2%)	168311 (46.8%)	7972 (2.2%)	9779 (2.7%)
Fla5 tuber	210093 (50.9%)	187131 (45.4%)	8590 (2.1%)	6689 (1.6%)
Fla1 soil	247254 (41.3%)	10302 (1.7%)	143882 (24.0%)	196872 (32.9%)
Fla2 soil	162489 (55.1%)	11897 (4.0%)	4306 (1.5%)	116293 (39.4%)
Fla3 soil	119365 (25.2%)	16375 (3.5%)	162328 (34.3%)	175012 (37.0%)
Fla4 soil	112709 (27.0%)	5260 (1.3%)	63251 (15.2%)	235662 (56.5%)
Fla5 soil	189964 (39.0%)	5627 (1.2%)	60762 (12.5%)	231281 (47.4%)

The percentages are relative to the number of the merged reads.

### Comparison of fungal communities by amplicon size distribution

**Figure 2:**
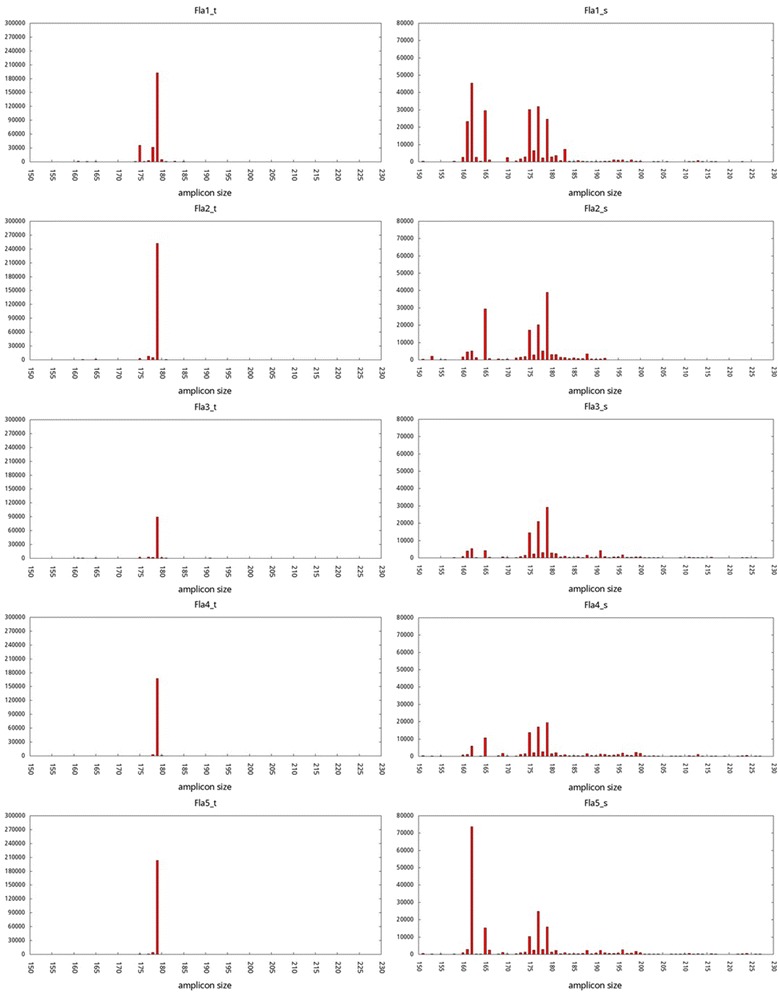
**Length distributions of merged reads, i.e., amplicons, of all tuber (left panel) and soil (right panel) samples.** Sample ID suffix: _t, tuber; _s, soil.

Our fungal specific primers were designed to amplify a variable region of LSU rDNA sequence. The amplified fungal 28S rDNA fragments were expected to be 167–218 bp in size. Most of the fungal amplicons of all the tuber samples were in the range of 175–180 bp and showed a single major peak at size of 179 bp (Figure 2). In contrast, the fungal amplicons of the soil samples were in the range of 162–200 bp and showed several major peaks. Compared to the tuber samples, the broader spectrum of amplicon sizes of the soil samples indicated greater fungal diversity in the soil surrounding the plants.

### Highly diversified fungal communities in tubers and the surrounding soil

**Figure 3:**
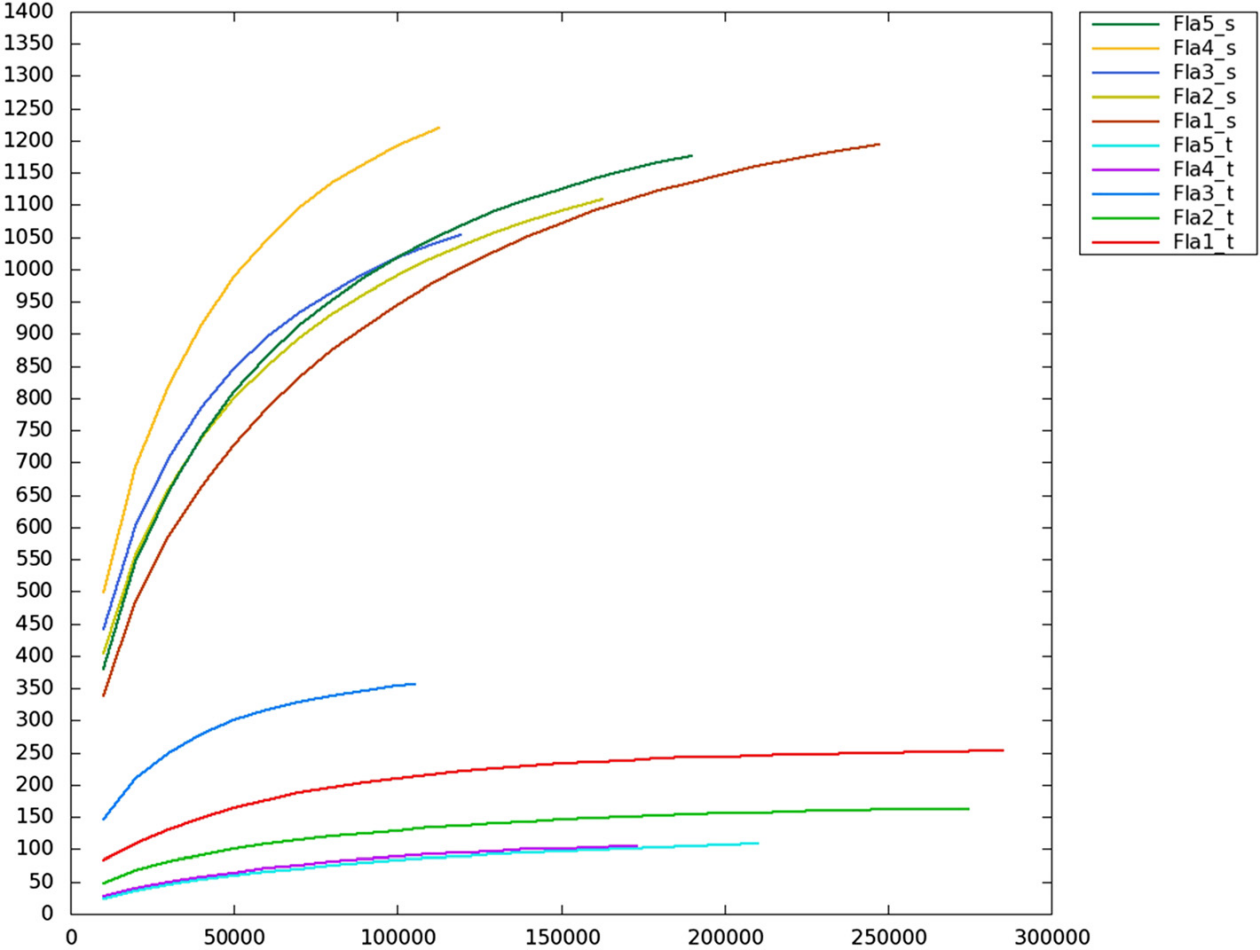
**OTU rarefaction curve: by randomly selecting smaller fractions of reads 100 times and counting the mean number of OTUs.**

It has been suggested that achlorophyllous orchids often show high specificity to their mycorrhizal fungi and mycorrhizal association may vary in photosynthetic orchids [3,5-7]. To study fungal diversity in the tubers and the surrounding soil, we examined the fungal communities by amplicon sequences. Before analyzing the fungal communities, we removed singletons and chimeric reads using the UPARSE pipeline (Methods). This removed 0.2-2.9% of the amplicons of tuber samples and 2.3-4.8% of the amplicons of the soil samples. For each sample, we clustered similar amplicon sequences into operational taxonomic units (OTUs) by UPARSE using a default setting of 97% sequence similarity. We found 82–353 and 945–1191 OTUs in the *G. flavilabella* tubers and the surrounding soil, respectively (Table 3). The numbers of OTUs were closer to saturation for the tuber samples than for the soil samples (Figure 3). Thus, the fungal diversity of the soil was underestimated. It is likely that we also have underestimated the fungi diversity in the tubers of *G. flavilabella* because some of the filtered singletons might be authentic. In any case, the clearly larger numbers of OTUs in the soil indicated more complex fungal communities in the soil (Table 3). Note that the majority of the OTUs in the tubers were not abundant (<1%, Additional file 1: Table S1). However, two to seven OTUs in the tubers occupied more than 1% of the fungal community, indicating concurrent association of several fungal species in the tubers of *G. flavilabella*.

**Table 3:** **Number of OTUs in the reads sampled from the tuber and soil data**

***Gastrodia*** **sample**	**No. of OTUs in 100,000 reads sampled from tuber data**	**No. of OTUs in 100,000 reads sampled from soil data**
Fla1	210.1 ± 4.4	945.6 ± 9.1
Fla2	129.2 ± 3.8	990.5 ± 7.5
Fla3	353.7 ± 1.6	1017.9 ± 5.2
Fla4	88.8 ± 3.3	1191.8 ± 4.5
Fla5	82.8 ± 3.5	1018.4 ± 8.7

### Fungal communities in the tubers and the soil are different

**Figure 4:**
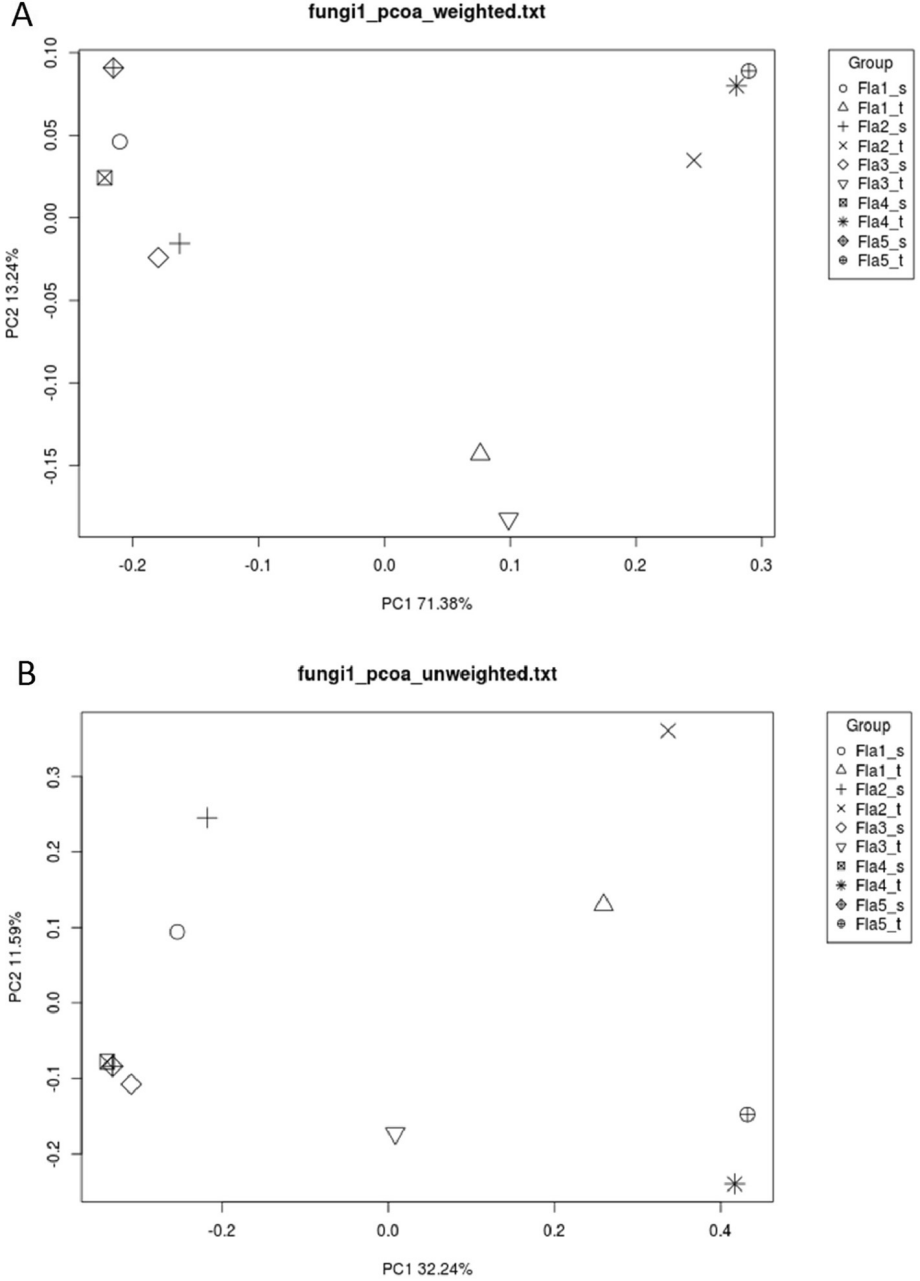
**Comparison of fungal communities: (a) weighted principal component analysis; the numbers of reads represented by OTU representatives were considered and (b) unweighted principal component analysis; the numbers of reads represented by OTU representatives were not considered.**

We examined if the fungal communities of the tubers are influenced by the fungal communities of the soil. To this end, the representative sequences of OTUs of all samples were gathered and the distances between the fungal communities were measured by UniFrac (Methods). The distances were visualized after principal component analysis (Figure 4). The first two principal axes of the weighted analysis, i.e., considering OTU abundance, captured 84.6% of the data variance. As shown in Figure 4a, all the soil samples formed a cluster, indicating a certain degree similarity of the fungal communities in the soil of the area. The fungal communities of all the tuber samples also formed a group, which could be divided into two subgroups: Fla1,3 and Fla2,4,5. This indicated different fungal communities in the plants of the two subgroups. Moreover, the fungal communities in nearby soil could change quickly with distance, as the fungal communities of the Fla4 and Fla5 soil samples (0.05 meter apart) did not cluster together. So far, we observed that (1) the fungal communities in the tubers were different from those in the soil, (2) fungal communities in tubers at different locations were similar and (3) the fungal communities in soil could change quickly with distance.

### Fungal communities and compositions in the tubers and the soil

**Figure 5:**
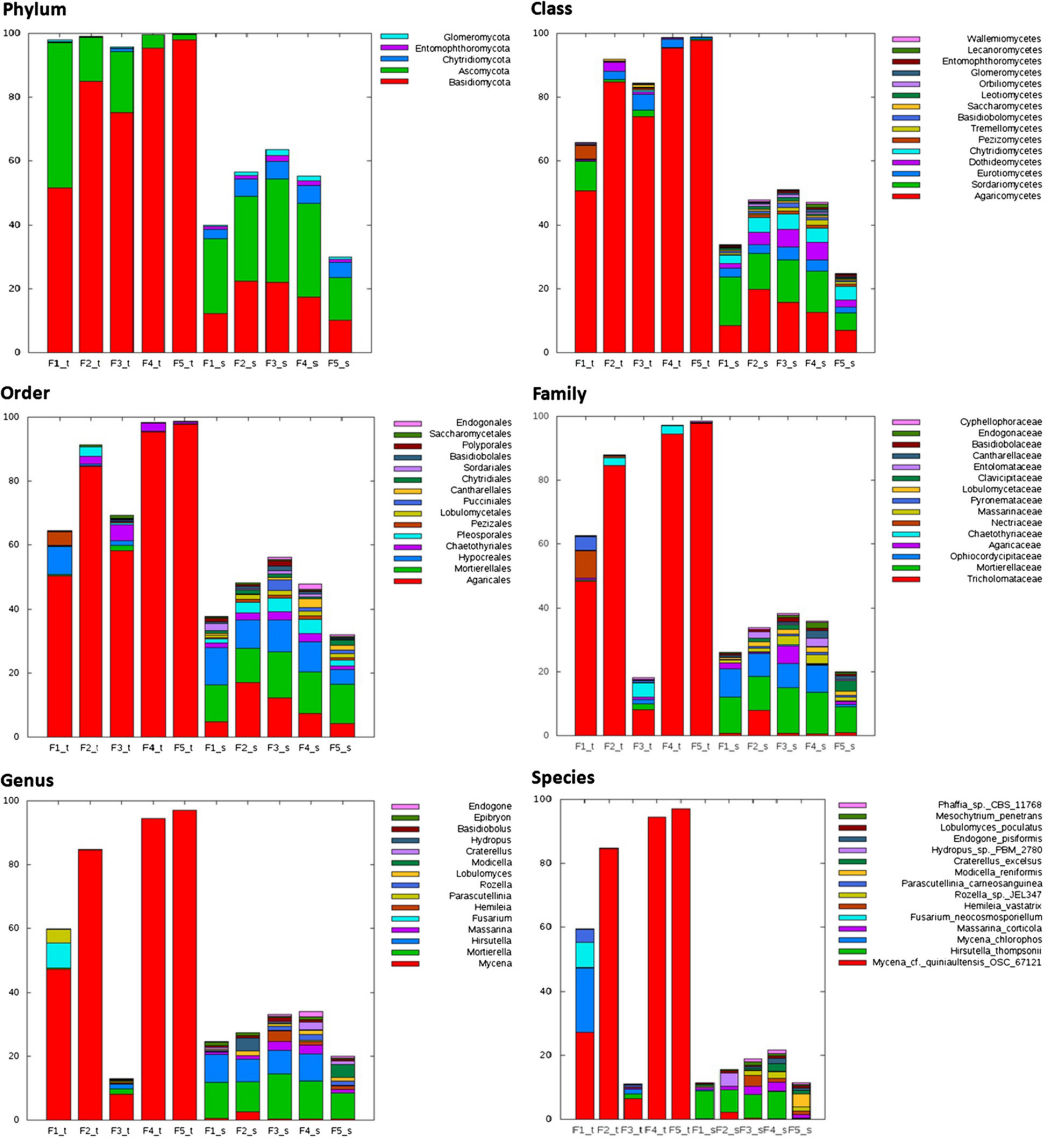
**Fungal compositions in tuber samples and the surrounding soil samples at six taxonomic levels.** At each level, we selected the 15 most abundant taxonomies (only the top 5 at the phylum level) based on the mean percentage across all samples and stacked them from bottom to top. The remaining classifications were lumped into “others”. The parts between the stack top and 100% were constituted by unclassified reads. Sample ID suffix: _t, tuber; _s, soil

To identify the fungal species in the tuber and soil samples, we examined the taxonomies of the OTUs of all the ten tuber and soil samples. We used a BLAST-based nearest neighbor approach for classifying the OTUs’ representative sequences. At the phylum level, the percentages (51.6-97.9%) of Basidiomycota fungi in all tuber samples were higher than those (10.2-22.4%) in the soil samples (Figure 5). Conversely, the percentages of Ascomycota fungi in all tuber samples except Fla1 were lower compared to the corresponding soil. In addition, the percentages of the next three abundant phyla in all tuber samples were lower than those in the soil samples. Most (98.1-99.9%) of the Basidiomycota fungi in the tuber samples were from the class Agaricomycetes, whereas the Ascomycota fungi split into several classes (e.g., Sordariomycetes, Eurotiomycetes, and Leotiomycetes). Similar trends were observed in the soil samples, but 12.0-30.9% of the Basidiomycota fungi in the soil did not belong to the Agaricomycetes. At the order level, the overall fungal compositions were similar to those at the phylum level. For example, most of the Agaricomycetes in the Fla tubers were Agaricales species. Such a nearly monocomponent of taxonomy at the lower taxonomic ranks continued to the family and genus levels for most of Fla tuber samples except for the Fla3 tuber sample, where most of the abundant OTUs were unclassified below the order level. These OTUs were unclassified beyond the order level mainly because the sequences were aligned equally well to more than one reference, which carried different taxonomic classifications (see Discussion).

At all taxonomic levels, especially the lower ones, the fungal composition between a tuber sample and the corresponding soil sample was apparently different (Figure 5). This and the following observation were consistent with Figure 4. First, the fungal communities of the Fla2, Fla4, and Fla5 tuber samples were more similar to each other than to the Fla1 and Fla3 tuber samples. Second, among the soil samples, Fla3 and Fla4 showed more similar fungal compositions compared to other samples. Third, the fungal communities of soil were more complex than those observed in the tubers. Compared to the tuber samples, the higher percentages of unclassified amplicons in the soil samples might indicate a large fraction of novel fungal species in the soil.

### *Mycena* species are the predominant fungi in *G. flavilabella*

The species closely related to *Mycena cf. quiniaultensis OSC 67121* constituted 84.6-97.0% of the fungi communities in the Fla2, Fla4, and Fla5 tuber samples (Figure 5). In the Fla1 tuber sample, 47.4% of fungi communities were *Mycena* species, of which 27.1% and 20.1% were closely related to *Mycena cf. quiniaultensis OSC 67121* and *Mycena chlorophos*, respectively. Thus, *Mycena* species were the predominant fungi in the *G. flavilabella*, except that only 8.1% of *Mycena* species were in the Fla3 tuber sample (Figure 5)*.* Note that the taxonomy of some abundant OTUs did not appear in Figure 5 when they were unclassified at the taxonomic level (Additional file 1: Table S1). Take Fla3 tuber sample for example, the most abundant OTU (48.9% of fungal reads) was classified as the Agaricales; however, it was perfectly aligned to 13 different *Mycena* species and 7 non-*Mycena* species. That is, the most abundant OTU in the Fla3 tuber might still be *Mycena* species, but they were surely distinct from *Mycena cf. quiniaultensis OSC 67121*. Our data also suggested that the predominance of *Mycena* fungi in the *G. flavilabella* tubers was not influenced by the fungal composition of the environment because the percentages (0.3-2.5%) of *Mycena* fungi in all soil samples were much lower than those in the tuber samples (Figure 5).

## Discussion

### Preferential association between *Mycena* fungi and *G. flavilabella*

Based on the clear distinction between the fungal communities in the *G. flavilabella* tubers and those in the surrounding soil (Figure 2, 3, and 4), we proposed that specific fungal communities had developed in the tubers of *G. flavilabella*. Moreover, the species closely related to *Mycena cf. quiniaultensis OSC 67121* (identity ≥ 98%) was the predominant fungus in *G. flavilabella* tubers because it constituted 84.6-97.0% of the fungi communities in the Fla2, Fla4, and Fla5 tuber samples, but only a small fraction in all the soil samples (Figure 4). Interestingly, some differences were observed among the tubers. For example, *Mycena* species closely related to *Mycena cf. quiniaultensis OSC 67121* or *Mycena chlorophos* were the top abundant fungi in the Fla1 tuber sample, while other *Mycena* and non-*Mycena* species were abundant in the Fla3 tuber sample*.* However, since they are all *Mycena* fungi, we concluded that adult *G. flavilabella* has a strong preference for association with *Mycena* fungi.

The highly diversified fungi in the tubers of *G. flavilabella* suggested that *G. flavilabella* does not have a strong defense mechanism to prevent fungal invasion and this perhaps reflects a fully mycoheterotrophic orchid’s nature to exploit any possible nutrient source. But why did the preference of *G. flavilabella* for *Mycena* fungi occur? It is possible that *Mycena* fungi were the first few fungi that invaded the tubers of *G. flavilabella* and *G. flavilabella* had since became dependent on *Mycena* fungi. It is also possible that the preference developed simply because *Mycena* fungi are saprotrophic and can provide *G. flavilabella* suitable nutrients. On the other hand, *G. flavilabella* might have changed its physiology to provide a stable environment for *Mycena* species or *G. flavilabella* might be able to stimulate the growth of *Mycena* species as has been observed in some mycorrhizal fungi [23].

### Association of saprotrophic fungi with *G. flavilabella*

To date, achlorophyllous orchids were the only plants reported to solely rely on the saprotrophic fungi for carbon and nutrition [4,16]. Most of the mycoheterotrophic plant associated mycorrhizal fungi also form symbiotic relationship with photosynthetic plants, so that many mycoheterotrophic plants may also obtain carbon and nutrients from surrounding photosynthetic plants through ectomycorrhizal fungi [24]. Ogura-Tsujita et al. reported that *G. confusa* is associated with several *Mycena* species and obtains carbon from these saprotrophic fungi but not from the surrounding photosynthetic plants [4]. It has been suggested that adult *G. elata* depends on *Armillaria* species for nutritional supply and can be cultivated without photosynthetic plants [13,14]. Now, there is more evidence that *Gastrodia* species is associated with litter- or wood-decaying fungi such as *Resinicium, Campanella* and *Marasmius* species for carbon and nutrients [15,16]. We found that *G. flavilabella* has preferential association with *Mycena* fungi, and possibly also with *Dictyopanus*, *Mycenoporella, Favolaschia*, *Panellus*, *Cruentomycena*, and *Resinomycena* fungi. Association of these litter- or wood-decaying saprotrophic fungi suggested that *G. flavilabella* is a fully mycoheterotrophic orchid that does not rely on photosynthetic plants. However, we cannot rule out that *G. flavilabella* also obtains nutrients from surrounding photosynthetic plants.

### Environmental factors might affect the fungal communities of tubers and the surrounding soil

Although the Fla4 and Fla5 plants were only ~0.05 meters apart (Figure 1), the fungal communities in the two soil samples were not the most similar among the soil samples, indicating that the fungal communities in soil could change quickly with distance (Figure 4a, 5 and Additional file 1: Table S1). However, the fungal communities of the Fla4 and Fla5 soil did cluster together in unweighted analysis, which considered only the identity but not the quantity of species (Figure 4b). Thus, the identities of microbes could be similar in a nearby region but their abundances could change quickly even in only 0.05 meters apart. As soil nutrient content could influence the abundance of soil microbes [25], we suspected that the slight difference of the microenvironment such as composition of nutrients could affect the abundance of fungi in the soil. It is likely that the environmental factors could also affect the fungal communities of *G. flavilabella* tubers. Indeed, environmental factors such as soil moisture, organic content, and total nitrogen content in Fla2, Fla4 and Fla5 soil samples were rather similar, which might explain the similarity among the fungal communities in Fla2, Fla4 and Fla5 tubers (Table 4 and Figure 4a). In addition, the clustering of fungal communities in the tubers was different from that of the soil (Figure 4a), indicating that the fungal community in a Fla tuber was not strongly influenced by the fungal community of the surrounding soil. Therefore, environmental factors such as water and nutrient content of the soil apparently have greater impacts on the fungal communities of tubers than the fungal communities of soil did.

**Table 4:** **The environmental factors of soil**

**soil sample**	**Soil moisture** ^**a**^ **(%)**	**Soil organic matter** ^**b**^ **(%)**	**Soil total- nitrogen content** ^**c**^ **(%)**
Fla1	51.9 ± 0.3	35.0 ± 0.9	1.32 ± 0.01
Fla2	32.4 ± 0.2	12.6 ± 0.4	0.45 ± 0.01
Fla3	57.3 ± 0.3	40.7 ± 0.5	1.35 ± 0.04
Fla4	36.6 ± 0	13.9 ± 0.2	0.46 ± 0.02
Fla5	36.3 ± 0.3	14.0 ± 0.3	0.47 ± 0.01

^a^Soil water/Soil mass.

^b^Organic mass/Soil mass.

^c^Total nitrogen/Soil mass.

### Metazoa in the orchid samples

Metazoa can infect plants with no mutual benefits, leading to plant parasitism. For example, the nematode *Meloidogyne incognita* can invade the roots of almost all cultivated plants [26,27] and induce the re-differentiation of root cells [28]. Many herbivore insects are also plant parasites and some arthropods can live within plants; e.g., phylloxera is an endo-parasite of grapes [29]. It is thus not peculiar to observe metazoan sequences in our tuber samples. Among the metazoan sequences in our Fla tuber samples, nematodes and arthropods were the two major phyla in most cases (Additional file 1: Figure S4b).

The metazoan sequences might be in part due to contamination from the surrounding soil. In this scenario, we expected the metazoan community in the Fla tubers to be, to some extent, similar to that in the surrounding soil. However, the Fla1, Fla4, and Fla5 tubers did not cluster with the surrounding soil in UniFrac analysis (Additional file 1 Figure S5a). When focusing on the identities of metazoa, i.e., unweighted analysis, only the Fla3 tuber and soil samples clustered together (Additional file 1: Figure S5b). Thus, contamination should not be a major concern. Interestingly, we did not observe a common pattern of metazoan compositions in the Fla tubers, indicating no host specificity for metazoans.

### LSU database

LSU sequences have been collected in the databases RDP [30] and SILVA [31]. However, we decided to curate our own LSU sequences because many LSU sequences in NCBI were not included in these databases. The RDP database (release 11) provides 62,860 fungal specific 28S rDNA sequences, so that they can not be used to classify non-fungi amplicons. In contrast, SILVA contains many non-fungal sequences but relatively few fungal sequences. Among the 39,412 LSU sequences in SILVA (release 115), 1,959, 5,600, and 4,145 belong to fungi, metazoa and viridiplantae, respectively. In contrast, we collected 268,614 LSU sequences, of which 120,617, 95,458, and 14,540 were fungi, metazoan and viridiplantae, respectively. Our more comprehensive LSU references allowed us to infer taxonomy with a higher accuracy. Note that some of our LSU sequences were not classified at all the seven taxonomic levels and some were only partial 28S rDNA sequences. This is perhaps one reason why other databases do not collect such sequences. Nevertheless, incomplete information was better than missing information and the partial 28S rDNA sequences were useful for our analysis.

### Taxonomy classification

Several computational tools, e.g., RDP classifier [30], Greengenes classifier [32] based on NAST alignments [33] and the BLAST-based method [34], have been proposed for taxonomic classification. The BLAST-based method was shown comparable to RDP classifier and Greengenes classifier in terms of internal consistency of taxonomic assignments, but was less sensitive than Greengenes [34]. At the time of this study, NAST was not available. Moreover, Greengenes was only available as a web-server and thus could not accommodate our large datasets. We had tried the RDP classifier, but found some practical challenges. First, the RDP classifier requires references to have complete taxonomy classifications from domain to genus. This immediately dismissed those perfectly aligned or highly identical references that had missing taxonomy information. Second, the reference sequences of RDP classifier were not comparable to our amplicons in length. Our BLAST-based nearest neighbor approach classified most (92.9-98.4%) of the good-quality merged tuber amplicons to fungi, metazoa or viridiplantae (Table 2). Although 32.9-56.5% of the soil amplicons could not be classified to fungi, metazoa or viridiplantae, those unclassified amplicons in the soil samples might correspond to the novel species in the soil.

### Confidence of taxonomy classification

Our own LSU references were still not sufficient for giving all the sequences the right classification. For example, most of the viridiplantae amplicons in all tuber samples were classified as “Viridiplantae; Streptophyta; Liliopsida; Asparagales; Orchidaceae; *Campylocentrum*; *Campylocentrum micranthum*” instead of being *G. flavilabella*. This was the result of no *Gastrodia* LSU sequence in the NCBI database. The classification, however, was reasonable because *Gastrodia* belongs to the Orchidaceae family. Moreover, the alignment identity of the corresponding OTU representative to the *Campylocentrum micranthum* reference was only 92.4% (data not shown).

The OTU representative classified as *Mycena cf. quiniaultensis OSC 67121* was 99.4% identical to the corresponding reference sequence (Additional file 1: Table S1). A similar observation was made for the OTU classified as *Mycena chlorophos*, the third most abundant OTU in the Fla1 tuber sample. In the Fla3 tuber sample, the three most abundant OTUs were classified as Agaricales, Agaricomycetes and Ascomycota fungi, respectively, but were aligned to multiple taxonomic groups at the family or higher level. For example, the most abundant OTU was classified as Agaricales fungi; however, the OTU representative was perfectly aligned to 13 different *Mycena* species and 7 non-*Mycena* species. Moreover, none of the best alignments were to the *Mycena cf. quiniaultensis OSC 67121*, indicating that the dominating *Mycena* species in the Fla3 tuber sample was different from those in other Fla tuber samples. The taxonomic classification for the second and third most abundant OTUs were Agaricomycetes and Ascomycota fungi; however, none of the best alignments was to the Tricholomataceae family, indicating non-*Mycena* species of these two OTUs. We emphasized again that our BLAST-based nearest neighbor approach to species classifications might not be 100 percent correct if the species sequence information in the database was missing or not correct. However, it is certain that the sequences classified differently, e.g., as *Mycena cf. quiniaultensis OSC 67121* and *Mycena chlorophos*, were distinct, thus truly representing different species.

## Conclusions

This is the first study using a NGS deep sequencing approach for identifying the fungal communities in the tubers of *G. flavilabella* and in the surrounding soil. We found highly diversified fungal communities in the tubers, and *Mycena* species were the predominant fungi in the tubers of *G. flavilabella*, indicating that the mycoheterotrophic *G. flavilabella* has a preferential association with saprotrophic *Mycena* fungi. So far, it has been shown that *Gastrodia* species such as *G. elata, G. confuse, G. similis* and *G. sesamoides* are associated with litter- or wood-decaying fungi such as *Armillaria, Mycena, Resinicium, Campanella* and *Marasmius* species for carbon and nutrients [4,13-16]. Finally, the environmental factors such as soil water and nutrient contents might have a greater impact on the fungal communities in the tubers than the fungal communities in the soil.

## Methods

### Sample preparation

In this study, we collected five tuber samples (Fla1-5) and five soil samples from their surrounding soil in the Hsitou area of Taiwan (Figure 1). Briefly, these samples were collected from an area with a 75 meter radius. Fla4 and Fla5 were located only about 0.05 meters apart from each other. Fla3 was ~150 meters away from Fla4 and Fla5. Fla1 and Fla2 were located between Fla3 and Fla4/Fla5 and the distance between Fla1 and Fla2 was ~12 meters. We individually collected one tuber of *G. flavilabella* and one surrounding soil sample (3 cm depth; 3 cm diameter) at each of five localities. The orchid tubers were surface-sterilized with 1% sodium hypochlorite for 30s and rinsed three times in sterile water for 30s to avoid contamination. The sterilized samples were then sagittal sectioned to about 1 g per fragment. Each plant surrounding soil was sieved to remove rocks and roots, and to break up the large soil aggregates. All of the orchid tuber and the surrounding soil samples were kept at −80°C for further analysis.

### Genomic DNA extraction

The CTAB extraction method [35,36] was used to extract genomic DNAs from the plant tuber samples (~1 g). Genomic DNAs from the surrounding soil samples were extracted by a commercial DNA extraction kit (PowerSoil DNA Isolation Kit, MoBio).

### PCR amplification of 28S rDNA markers

For each tuber or soil sample, the PCR reaction was carried out in a 50 μl reaction with 1ul template DNA, 0.5 unit of GoTaq polymerase (Promega, US), 5 μl of 10X PCR buffer, 5 μl of 1X dNTP mix (2.5 μM each dNTP), 3 μl of MgCl_2_ (25 mM), and 4 μl of each fungal-specific primer (3 μM). The PCRs were run with 35 cycles of 95°C for 30s, 56.2°C for 30s, and 72°C for 30s, and a final extension of 72°C for 10 min and the PCR products were purified with QiAquick PCR purification kit (Qiagen) following manufacture’s instruction. We designed a set of fungal specific LSU rDNA primers, AACACGGACCAAGGAGTC (forward) and CAGGCATAGTTCACCATCTT (reverse) which target a LSU region that is conserved in fungi but not in other organisms and amplify a variable region of the LSU rDNA. The amplified LSU regions are expected to be 167–218 bp in size, which can be covered by the Illumina paired-end reads.

### Illumina paired-end (PE) sequencing

DNA sequencing was carried out on Illumina HiSeq 2500 platform (the Fla2 sample was on HiSeq 2000) at Yourgene Biosciences, Taiwan, following the manufacturer’s protocols. Briefly, amplicon DNA was A-tailed using the polymerase activity of Klenow fragment. Indexed adapters were then ligated to the DNA fragments by DNA ligase followed by PCR reaction of 10 to 18 cycles to enrich the adapter-modified DNA fragments. Before sequencing, the libraries were validated by QPCR, Expersion and Qubit.

### Data processing

For each Illumina PE library, we first aligned the 28S primer sequences to all reads using BLAST (v2.2.29+, options: −word_size 5 -evalue 0.001). A read was considered qualified if a whole primer was aligned on the positive strand of the read and the primer was aligned only once to the read. It was possible that both forward and reverse primers appeared on a read. In that case, the mate-read was also required contained both primers if the mate-read was long enough. For each qualified read, we trimmed the segment outside primer from the read if there was any. A PE was qualified if both reads were qualified and the corresponding primers formed a pair. The paired reads of all qualified PE were further merged by FLASH [37] (v1.2.7) with default parameters (requiring ≥10 bp overlap) and short merged reads (<100 bp) were discarded. High-quality merged reads were then selected for analysis. Specifically, we scanned each merged read using a window size of 5 bp and required the average quality to be at least 30 throughout the read. For OTU analysis by UPARSE, read orientation was adjusted so that the forward primer was at the 5′ end of all merged reads.

### Large subunit rDNA references

We obtained from NCBI [38] non-redundant nucleotides (nt, last modified on 2014.03.11) and parsed out the large subunit (LSU) rDNA sequences. Specifically, we kept the sequences whose description ($d) contained the keywords “large”, “LSU”, “28S”, and “rRNA” (perl script: ($d = ~/[Ll]arge/ || $d = ~/[(nr)\s\(\-)LSU[\s\],]/ || $d = ~/[\s\(\/\-)2[3-8]S[\s\-]/] && ($d = ~/r[DR]NA/ || ($d = ~/ribosomal/ && $d = ~/[DR]NA/)). The sequences whose description contained the keyword “mRNA” or “spacer” were further excluded. The taxonomies at seven levels (domain, phylum, class, order, family, genus, and species) of the retained LSU rDNA sequences were then assign based on the gene ID. The mappings between gene ID and taxonomy ID were obtained from the file “gi_taxid_nucl.dmp” in the NCBI Taxonomy database (last modified on 2014.03.10). We parsed the two files “name.dmp” and “nodes.dmp” in the same database for the full lineages of all taxonomy IDs. Note that the parsed LSU rDNAs came from several domains, including fungi, metazoa, viridiplantae, etc. Also note that the taxonomy classifications of many sequences were null at some taxonomy levels, which were considered as unclassified at those levels.

### Taxonomy classification

To determine the taxonomy of the high-quality merged reads, we aligned the reads to the LSU rRNA references using MegaBLAST [39,40] (v2.2.29+, option: −word_size 16 -evalue 1e-10). For each read, the top alignment(s) with the lowest E-value, i.e., a nearest neighbor approach, was used for annotation. The corresponding taxonomy lineages, which could be more than one, were modified as follows. First, the lineages containing the keyword “uncultured” or “fungal_sp” were treated as unclassified lineages. Unclassified lineages were discarded if classified one(s) existed. Second, the lineages with the most complete classifications were selected. Third, the classification at a taxonomy level was considered unclassified if there was more than one classification. Based on the taxonomy, we split the reads by domain.

### OTU analysis

For each sample, we used the UPARSE pipeline [41] (v7.0.1090) to cluster the fungal reads into operational taxonomic units (OTUs) as follows. First, we took only the unique reads and recorded the read counts (usearch –derep_fulllength –sizeout). Second, the unique reads were sorted by read count and the singletons were removed (−sortbysize –minsize 2). Third, OTU clustering was performed (−cluster_otus). Fourth, chimeric OTUs were further filtered using NCBI LSU sequences as references (−uchime_ref –db ncbilsu.fa –strand plus –nonchimeras). Lastly, the unique reads were re-assigned to the OTUs with an identity cutoff 0.97 (−usearch_global –starnd plus –id 0.97) [42,43]. The statistics of OTUs were then obtained and the OTU representative sequences were collected for comparing fungal communities.

### Comparison of fungal communities

To compare fungal communities, the OTU representatives of all tuber and soil samples were first collected for constructing a phylogenetic tree using the mothur package [44] (v1.32, commands: align.seqs, dist.seqs, clearcut). Mothur requires aligned reference sequences for tree construction. To prepare the references, we extracted the fungal LSU rRNA references and ran e-PCR [45] (v2.3.11, fahash option: −w 3, re-PCR option: −n 2 -g 2 1–300) to locate binding sites for our 28S primers. The fungal LSU rDNAs containing the binding sites of both primers were kept and the corresponding segments between the two binding sites were extracted. We then used Clustal Omega [46] (v1.2, default parameters) to obtain the multiple sequence alignment of the extracted segments, which served as the curated references for tree construction. Based on the tree, the distances between samples were calculated by Fast UniFrac [47] (v1.5.3). We did both weighted (i.e., considering the numbers of reads represented by OTU representatives) and un-weighted principle component and clustering analyses. The distances between fungal communities were visualized using the R package [48] (v3.0.1).

To show the fungal compositions of a sample, we re-assigned taxonomy to the OTU representatives as above and calculated their fractions. Note that we used only the fungal LSU sequences as references. The results of all samples at different taxonomy levels were shown in stacked histograms.

### Availability of supporting data

The merged PE reads of the ten tuber and soil samples were deposited in NCBI SRA database under the SRA ID SRP054374. Detail information of DNA sequences along with the supporting Tables and Figures were included in the Additional file 1.

## Additional file

Additional file 1:
**Supporting data for highly diversified fungi are associated with the achlorophyllous orchid** ***Gastrodia flavilabella.***

## References

[1] Rasmussen HN (1995). Terrestrial Orchids, from Seed To Mycotrophic Plant.

[2] Leake JR (2004). Myco-heterotroph/epiparasitic plant interactions with ectomycorrhizal and arbuscular mycorrhizal fungi. Curr Opin Plant Biol.

[3] Barrett CF, Freudenstein JV, Taylor DL, Koljalg U (2010). Rangewide analysis of fungal associations in the fully mycoheterotrophic *Corallorhiza striata* complex (Orchidaceae) reveals extreme specificity on ectomycorrhizal *Tomentella* (Thelephoraceae) across North America. Am J Bot.

[4] Ogura-Tsujita Y, Gebauer G, Hashimoto T, Umata H, Yukawa T (2009). Evidence for novel and specialized mycorrhizal parasitism: the orchid *Gastrodia confusa* gains carbon from saprotrophic *Mycena*. Proc Biol Sci.

[5] Ogura-Tsujita Y, Yukawa T (2008). High mycorrhizal specificity in a widespread mycoheterotrophic plant, *Eulophia zollingeri* (Orchidaceae). Am J Bot.

[6] Jacquemyn H, Honnay O, Cammue BP, Brys R, Lievens B (2010). Low specificity and nested subset structure characterize mycorrhizal associations in five closely related species of the genus *Orchis*. Mol Ecol.

[7] Shefferson RP, Weiss M, Kull T, Taylor DL (2005). High specificity generally characterizes mycorrhizal association in rare lady’s slipper orchids, genus *Cypripedium*. Mol Ecol.

[8] Hsu T-C (2008). Master.

[9] Xu J-T, Guo S-X (2000). Retrospect on the research of the cultivation of *Gastrodia elata* Bl, a rare traditional Chinese medicine. Chin Med J (Engl).

[10] Fan L, Guo S-X (1999). Interaction between protocorms of *Gastrodia elata* (orchidaceae) and *Mycena dendrobii* in symbiotic germination. Mycosystema.

[11] Fan L, Guo S-X, Xiao P-G (2001). Interaction between protocorms of *Gastrodia elata* (orchidaceae) and *Mycena anoectochila* during symbiotic germination. Mycosystema.

[12] Kim YI, Chang KJ, Ka KH, Hur H, Hong IP, Shim JO (2006). Seed Germination of *Gastrodia elata* Using Symbiotic Fungi, *Mycena osmundicola*. Mycobiology.

[13] Cha JY, Igarashi T (1995). *Armillari*a species associated with *Gastrodia elata* in Japan. Eur J Forest Pathol.

[14] Sekizaki H, Kuninaga S, Yamamoto M, Asazu SN, Sawa S, Kojoma M (2008). Identification of *Armillaria nabsnona* in gastrodia tubers. Biol Pharm Bull.

[15] Dearnaley JDW, Bougoure JJ (2010). Isotopic and molecular evidence for saprotrophic Marasmiaceae mycobionts in rhizomes of *Gastrodia sesamoides*. Fungal Ecol.

[16] Martos F, Dulormne M, Pailler T, Bonfante P, Faccio A, Fournel J (2009). Independent recruitment of saprotrophic fungi as mycorrhizal partners by tropical achlorophyllous orchids. New Phytol.

[17] Lievens B, van Kerckhove S, Juste A, Cammue BP, Honnay O, Jacquemyn H (2010). From extensive clone libraries to comprehensive DNA arrays for the efficient and simultaneous detection and identification of orchid mycorrhizal fungi. J Microbiol Methods.

[18] Martos F, Munoz F, Pailler T, Kottke I, Gonneau C, Selosse MA (2012). The role of epiphytism in architecture and evolutionary constraint within mycorrhizal networks of tropical orchids. Mol Ecol.

[19] Roy M, Yagame T, Yamato M, Iwase K, Heinz C, Faccio A (2009). Ectomycorrhizal Inocybe species associate with the mycoheterotrophic orchid *Epipogium aphyllum* but not its asexual propagules. Ann Bot.

[20] Xing X, Ma X, Deng Z, Chen J, Wu F, Guo S (2013). Specificity and preference of mycorrhizal associations in two species of the genus *Dendrobium* (Orchidaceae). Mycorrhiza.

[21] Taylor DL, Bruns TD (1999). Community structure of ectomycorrhizal fungi in a *Pinus muricata* forest: minimal overlap between the mature forest and resistant propagule communities. Mol Ecol.

[22] McCormick MK, Whigham DF, O’Neill JP, Becker JJ, Werner S, Rasmussen HN (2009). Abundance and distribution of *Corallorhiza odontorhiza* reflect variations in climate and ectomycorrhizae. Ecol Monogr.

[23] Bidartondo MI (2005). The evolutionary ecology of myco-heterotrophy. New Phytol.

[24] Leake JR (2005). Plants parasitic on fungi: unearthing the fungi in myco-heterotrophs and debunking the ‘saprophytic’ plant myth. Mycologist.

[25] Koorem K, Gazol A, Opik M, Moora M, Saks U, Uibopuu A (2014). Soil nutrient content influences the abundance of soil microbes but not plant biomass at the small-scale. PLoS One.

[26] Blaxter ML (2003). Nematoda: genes, genomes and the evolution of parasitism. Adv Parasitol.

[27] Trudgill DL, Blok VC (2001). Apomictic, polyphagous root-knot nematodes: exceptionally successful and damaging biotrophic root pathogens. Annu Rev Phytopathol.

[28] Caillaud MC, Lecomte P, Jammes F, Quentin M, Pagnotta S, Andrio E (2008). MAP65-3 microtubule-associated protein is essential for nematode-induced giant cell ontogenesis in Arabidopsis. Plant Cell.

[29] Granett J, Walker MA, Kocsis L, Omer AD (2001). Biology and management of grape phylloxera. Annu Rev Entomol.

[30] Cole JR, Wang Q, Fish JA, Chai B, McGarrell DM, Sun Y (2014). Ribosomal Database Project: data and tools for high throughput rRNA analysis. Nucleic Acids Res.

[31] Quast C, Pruesse E, Yilmaz P, Gerken J, Schweer T, Yarza P (2013). The SILVA ribosomal RNA gene database project: improved data processing and web-based tools. Nucleic Acids Res.

[32] DeSantis TZ, Hugenholtz P, Larsen N, Rojas M, Brodie EL, Keller K (2006). Greengenes, a chimera-checked 16S rRNA gene database and workbench compatible with ARB. Appl Environ Microbiol.

[33] DeSantis TZ, Hugenholtz P, Keller K, Brodie EL, Larsen N, Piceno YM (2006). a multiple sequence alignment server for comparative analysis of 16S rRNA genes. Nucleic Acids Res.

[34] Liu Z, DeSantis TZ, Andersen GL, Knight R (2008). Accurate taxonomy assignments from 16S rRNA sequences produced by highly parallel pyrosequencers. Nucleic Acids Res.

[35] Gardes M, Bruns TD (1993). ITS primers with enhanced specificity for basidiomycetes–application to the identification of mycorrhizae and rusts. Mol Ecol.

[36] Murray MG, Thompson WF (1980). Rapid isolation of high molecular weight plant DNA. Nucleic Acids Res.

[37] Magoc T, Salzberg SL (2011). FLASH: fast length adjustment of short reads to improve genome assemblies. Bioinformatics.

[38] Coordinators NR (2014). Database resources of the National Center for Biotechnology Information. Nucleic Acids Res.

[39] Altschul SF, Gish W, Miller W, Myers EW, Lipman DJ (1990). Basic local alignment search tool. J Mol Biol.

[40] Morgulis A, Coulouris G, Raytselis Y, Madden TL, Agarwala R, Schaffer AA (2008). Database indexing for production MegaBLAST searches. Bioinformatics.

[41] Edgar RC (2013). UPARSE: highly accurate OTU sequences from microbial amplicon reads. Nat Methods.

[42] Brown SP, Rigdon-Huss AR, Jumpponen A (2014). Analyses of ITS and LSU gene regions provide congruent results on fungal community responses. Fungal Ecol.

[43] Weber CF, Vilgalys R, Kuske CR (2013). Changes in Fungal Community Composition in Response to Elevated Atmospheric CO2 and Nitrogen Fertilization Varies with Soil Horizon. Front Microbiol.

[44] Schloss PD, Westcott SL, Ryabin T, Hall JR, Hartmann M, Hollister EB (2009). Introducing mothur: open-source, platform-independent, community-supported software for describing and comparing microbial communities. Appl Environ Microbiol.

[45] Rotmistrovsky K, Jang W, Schuler GD (2004). A web server for performing electronic PCR. Nucleic Acids Res.

[46] Sievers F, Wilm A, Dineen D, Gibson TJ, Karplus K, Li W (2011). Fast, scalable generation of high-quality protein multiple sequence alignments using Clustal Omega. Mol Syst Biol.

[47] Hamady M, Lozupone C, Knight R (2010). Fast UniFrac: facilitating high-throughput phylogenetic analyses of microbial communities including analysis of pyrosequencing and PhyloChip data. ISME J.

[48] Team RC (2013). R: A language and environment for statistical computing.

