# Supplementary material for: Highly diversified fungi are associated with the achlorophyllous orchid Gastrodia flavilabella
Source: BMC Genomics. 2015 Mar 14;16(1):185. doi: 10.1186/s12864-015-1422-7 (PMC4371811; doi:10.1186/s12864-015-1422-7)
Supplement: Additional file 1: — Supporting data for highly diversified fungi are associated with the achlorophyllous orchid Gastrodia flavilabella. [file 12864_2015_1422_MOESM1_ESM.doc]

**Supplementary Materials**

Tsunglin Liu, Ching-Min Li, Yue-Lun Han, Tzen-Yuh Chiang, Yu-Chung Chiang, Huang-Mo Sung

**DNA sequence information**

The merged PE reads of the ten tuber and soil samples were deposited in NCBI SRA database under the SRA ID SRP054374. Accession numbers of all samples are listed below.

| Sample | Accession number |
| --- | --- |
| Fla1_tuber | SRR1800249 |
| Fla2_tuber | SRR1800328 |
| Fla3_tuber | SRR1800398 |
| Fla4_tuber | SRR1800413 |
| Fla5_tuber | SRR1800424 |
| Fla1_soil | SRR1800434 |
| Fla2_soil | SRR1800442 |
| Fla3_soil | SRR1800447 |
| Fla4_soil | SRR1800453 |
| Fla5_soil | SRR1800455 |

**Supplementary Tables**

Table S1. Taxonomy of top abundant OTUs in domain fungi. Both “---” and “unc” indicate unclassified species. The former is the result of missing classification in the aligned reference while the later indicate multiple classifications in the aligned references.

| OTU | % | Identity; Coverage(%) | Taxonomy |
| --- | --- | --- | --- |
| Fla1_t | | | |
| 1 | 27.08 | 99.44;100.00 | Fungi;Basidiomycota;Agaricomycetes;Agaricales;Tricholomataceae;Mycena;Mycena_cf._quiniaultensis_OSC_67121 |
| 2 | 20.44 | 99.44;100.00 | Fungi;Ascomycota;unc;unc;unc;unc;unc |
| 3 | 20.08 | 99.44;100.00 | Fungi;Basidiomycota;Agaricomycetes;Agaricales;Tricholomataceae;Mycena;Mycena_chlorophos |
| 4 | 9.91 | 99.44;100.00 | Fungi;Ascomycota;unc;unc;unc;unc;unc |
| 5 | 7.79 | 98.86;100.00 | Fungi;Ascomycota;Sordariomycetes;Hypocreales;Nectriaceae;Fusarium;Fusarium_neocosmosporiellum |
| 6 | 3.25 | 83.24;100.00 | Fungi;Ascomycota;Pezizomycetes;Pezizales;Pyronemataceae;Parascutellinia;Parascutellinia_carneosanguinea |
| 7 | 1.13 | 96.11;100.00 | Fungi;Basidiomycota;Agaricomycetes;Agaricales;unc;unc;unc |
| 8 | 0.87 | 82.26;100.00 | Fungi;Ascomycota;Pezizomycetes;Pezizales;Pyronemataceae;Parascutellinia;Parascutellinia_carneosanguinea |
| 9 | 0.69 | 100.00;100.00 | Fungi;Ascomycota;Sordariomycetes;Hypocreales;Nectriaceae;Calonectria;Calonectria_mexicana |
| 10 | 0.62 | 90.81;100.00 | Fungi;Basidiomycota;---;---;---;---;--- |
| Fla2_t | | | |
| 1 | 84.28 | 99.44;100.00 | Fungi;Basidiomycota;Agaricomycetes;Agaricales;Tricholomataceae;Mycena;Mycena_cf._quiniaultensis_OSC_67121 |
| 2 | 5.31 | 100.00;100.00 | Fungi;Ascomycota;unc;unc;unc;unc;unc |
| 3 | 2.61 | 100.00;98.31 | Fungi;Ascomycota;Dothideomycetes;Pleosporales;unc;unc;unc |
| 4 | 2.42 | 99.43;98.32 | Fungi;Ascomycota;Eurotiomycetes;Chaetothyriales;Chaetothyriaceae;unc;unc |
| 5 | 1.47 | 99.44;100.00 | Fungi;Ascomycota;unc;unc;unc;unc;unc |
| 6 | 0.55 | 100.00;100.00 | Fungi;Ascomycota;Sordariomycetes;Hypocreales;Nectriaceae;Calonectria;Calonectria_mexicana |
| 7 | 0.42 | 80.61;100.00 | Fungi;---;---;---;---;---;--- |
| 8 | 0.32 | 96.09;100.00 | Fungi;Basidiomycota;Agaricomycetes;Agaricales;Tricholomataceae;Mycena;Mycena_cf._quiniaultensis_OSC_67121 |
| 9 | 0.28 | 100.00;100.00 | Fungi;Ascomycota;Saccharomycetes;Saccharomycetales;Saccharomycetaceae;Kluyveromyces;unc |
| 10 | 0.22 | 100.00;100.00 | Fungi;Ascomycota;Saccharomycetes;Saccharomycetales;Saccharomycetaceae;Saccharomyces;unc |
| Fla3_t | | | |
| 1 | 48.90 | 100.00;100.00 | Fungi;Basidiomycota;Agaricomycetes;Agaricales;unc;unc;unc |
| 2 | 15.30 | 98.32;100.00 | Fungi;Basidiomycota;Agaricomycetes;unc;unc;unc;unc |
| 3 | 9.24 | 100.00;100.00 | Fungi;Ascomycota;unc;unc;unc;unc;unc |
| 4 | 6.52 | 99.44;100.00 | Fungi;Basidiomycota;Agaricomycetes;Agaricales;Tricholomataceae;Mycena;Mycena_cf._quiniaultensis_OSC_67121 |
| 5 | 4.30 | 99.43;98.32 | Fungi;Ascomycota;Eurotiomycetes;Chaetothyriales;Chaetothyriaceae;unc;unc |
| 6 | 1.65 | 100.00;100.00 | Fungi;---;---;Mortierellales;Mortierellaceae;Mortierella;unc |
| 7 | 1.59 | 98.32;100.00 | Fungi;Basidiomycota;Agaricomycetes;Agaricales;Tricholomataceae;Mycena;Mycena_chlorophos |
| 8 | 1.38 | 99.43;100.00 | Fungi;Ascomycota;Sordariomycetes;Hypocreales;Ophiocordycipitaceae;Hirsutella;Hirsutella_thompsonii |
| 9 | 0.75 | 99.44;100.00 | Fungi;Basidiomycota;Agaricomycetes;Agaricales;Agaricaceae;unc;unc |
| 10 | 0.49 | 100.00;100.00 | Fungi;Ascomycota;Eurotiomycetes;Chaetothyriales;Cyphellophoraceae;Cyphellophora;unc |
| Fla4_t | | | |
| 1 | 92.62 | 99.44;100.00 | Fungi;Basidiomycota;Agaricomycetes;Agaricales;Tricholomataceae;Mycena;Mycena_cf._quiniaultensis_OSC_67121 |
| 2 | 2.52 | 99.43;98.32 | Fungi;Ascomycota;Eurotiomycetes;Chaetothyriales;Chaetothyriaceae;unc;unc |
| 3 | 1.76 | 95.53;100.00 | Fungi;Basidiomycota;Agaricomycetes;Agaricales;Tricholomataceae;Mycena;Mycena_cf._quiniaultensis_OSC_67121 |
| 4 | 0.90 | 100.00;100.00 | Fungi;Basidiomycota;Agaricomycetes;Agaricales;unc;unc;unc |
| 5 | 0.65 | 100.00;100.00 | Fungi;Ascomycota;unc;unc;unc;unc;unc |
| 6 | 0.33 | 97.77;100.00 | Fungi;Ascomycota;Dothideomycetes;Capnodiales;Mycosphaerellaceae;---;Mycosphaerellaceae_sp._SD-01 |
| 7 | 0.15 | 99.44;100.00 | Fungi;Ascomycota;unc;unc;unc;unc;unc |
| 8 | 0.14 | 100.00;100.00 | Fungi;Ascomycota;Sordariomycetes;Hypocreales;Nectriaceae;Calonectria;Calonectria_mexicana |
| 9 | 0.09 | 98.88;100.00 | Fungi;Ascomycota;Eurotiomycetes;Chaetothyriales;Cyphellophoraceae;Cyphellophora;unc |
| 10 | 0.08 | 85.80;100.00 | Fungi;---;---;---;---;---;--- |
| Fla5_t | | | |
| 1 | 95.19 | 99.44;100.00 | Fungi;Basidiomycota;Agaricomycetes;Agaricales;Tricholomataceae;Mycena;Mycena_cf._quiniaultensis_OSC_67121 |
| 2 | 1.82 | 95.53;100.00 | Fungi;Basidiomycota;Agaricomycetes;Agaricales;Tricholomataceae;Mycena;Mycena_cf._quiniaultensis_OSC_67121 |
| 3 | 0.70 | 100.00;100.00 | Fungi;Basidiomycota;Agaricomycetes;Agaricales;Tricholomataceae;Poromycena;Poromycena_sp._JM98/128 |
| 4 | 0.56 | 100.00;100.00 | Fungi;Ascomycota;unc;unc;unc;unc;unc |
| 5 | 0.49 | 98.88;100.00 | Fungi;Ascomycota;Eurotiomycetes;Chaetothyriales;Cyphellophoraceae;Cyphellophora;unc |
| 6 | 0.23 | 100.00;100.00 | Fungi;Ascomycota;Sordariomycetes;Hypocreales;Nectriaceae;Calonectria;Calonectria_mexicana |
| 7 | 0.18 | 99.44;100.00 | Fungi;Ascomycota;unc;unc;unc;unc;unc |
| 8 | 0.10 | 99.43;98.32 | Fungi;Ascomycota;Eurotiomycetes;Chaetothyriales;Chaetothyriaceae;unc;unc |
| 9 | 0.07 | 89.51;100.00 | Fungi;---;---;---;---;---;--- |
| 10 | 0.06 | 83.52;100.00 | Fungi;Chytridiomycota;Chytridiomycetes;unc;unc;unc;unc |
| Fla1_s | | | |
| 1 | 10.74 | 98.15;100.00 | Fungi;---;---;---;---;---;--- |
| 2 | 10.61 | 100.00;100.00 | Fungi;---;---;Mortierellales;Mortierellaceae;Mortierella;unc |
| 3 | 8.77 | 99.43;100.00 | Fungi;Ascomycota;Sordariomycetes;Hypocreales;Ophiocordycipitaceae;Hirsutella;Hirsutella_thompsonii |
| 4 | 8.03 | 88.27;100.00 | Fungi;---;---;---;---;---;--- |
| 5 | 7.93 | 80.61;100.00 | Fungi;---;---;---;---;---;--- |
| 6 | 4.58 | 82.42;100.00 | Fungi;---;---;---;---;---;--- |
| 7 | 3.95 | 96.30;100.00 | Fungi;---;---;---;---;---;--- |
| 8 | 2.59 | 90.81;100.00 | Fungi;Basidiomycota;---;---;---;---;--- |
| 9 | 2.17 | 92.59;100.00 | Fungi;---;---;---;---;---;--- |
| 10 | 2.16 | 100.00;98.29 | Fungi;Ascomycota;Sordariomycetes;Sordariales;unc;unc;unc |
| Fla2_s | | | |
| 1 | 13.61 | 80.61;100.00 | Fungi;---;---;---;---;---;--- |
| 2 | 9.36 | 100.00;100.00 | Fungi;---;---;Mortierellales;Mortierellaceae;Mortierella;unc |
| 3 | 7.03 | 99.43;100.00 | Fungi;Ascomycota;Sordariomycetes;Hypocreales;Ophiocordycipitaceae;Hirsutella;Hirsutella_thompsonii |
| 4 | 5.23 | 82.42;100.00 | Fungi;---;---;---;---;---;--- |
| 5 | 4.06 | 99.44;100.00 | Fungi;Basidiomycota;Agaricomycetes;Agaricales;Tricholomataceae;Hydropus;Hydropus_sp._PBM_2780 |
| 6 | 3.54 | 100.00;100.00 | Fungi;Basidiomycota;Agaricomycetes;Agaricales;unc;unc;unc |
| 7 | 2.21 | 99.44;100.00 | Fungi;Basidiomycota;Agaricomycetes;Agaricales;Tricholomataceae;Mycena;Mycena_cf._quiniaultensis_OSC_67121 |
| 8 | 1.92 | 83.51;100.00 | Fungi;Ascomycota;---;---;---;---;--- |
| 9 | 1.66 | 98.88;100.00 | Fungi;Basidiomycota;Agaricomycetes;Agaricales;Entolomataceae;Entoloma;unc |
| 10 | 1.61 | 100.00;100.00 | Fungi;Ascomycota;unc;unc;unc;unc;unc |
| Fla3_s | | | |
| 1 | 13.14 | 100.00;100.00 | Fungi;---;---;Mortierellales;Mortierellaceae;Mortierella;unc |
| 2 | 7.41 | 99.43;100.00 | Fungi;Ascomycota;Sordariomycetes;Hypocreales;Ophiocordycipitaceae;Hirsutella;Hirsutella_thompsonii |
| 3 | 5.46 | 99.44;100.00 | Fungi;Basidiomycota;Agaricomycetes;Agaricales;Agaricaceae;unc;unc |
| 4 | 3.85 | 100.00;100.00 | Fungi;Basidiomycota;Agaricomycetes;Agaricales;unc;unc;unc |
| 5 | 3.41 | 80.61;100.00 | Fungi;---;---;---;---;---;--- |
| 6 | 3.34 | 81.03;100.00 | Fungi;Basidiomycota;---;Pucciniales;---;Hemileia;Hemileia_vastatrix |
| 7 | 2.76 | 88.82;100.00 | Fungi;---;---;---;---;---;--- |
| 8 | 2.68 | 99.44;100.00 | Fungi;Ascomycota;Dothideomycetes;Pleosporales;Massarinaceae;Massarina;Massarina_corticola |
| 9 | 1.59 | 100.00;100.00 | Fungi;Ascomycota;unc;unc;unc;unc;unc |
| 10 | 1.55 | 98.86;100.00 | Fungi;Ascomycota;Sordariomycetes;Hypocreales;Clavicipitaceae;unc;unc |
| Fla4_s | | | |
| 1 | 11.53 | 100.00;100.00 | Fungi;---;---;Mortierellales;Mortierellaceae;Mortierella;unc |
| 2 | 9.14 | 80.61;100.00 | Fungi;---;---;---;---;---;--- |
| 3 | 8.43 | 99.43;100.00 | Fungi;Ascomycota;Sordariomycetes;Hypocreales;Ophiocordycipitaceae;Hirsutella;Hirsutella_thompsonii |
| 4 | 2.77 | 99.44;100.00 | Fungi;Ascomycota;Dothideomycetes;Pleosporales;Massarinaceae;Massarina;Massarina_corticola |
| 5 | 2.61 | 92.59;100.00 | Fungi;---;---;---;---;---;--- |
| 6 | 2.44 | 98.88;100.00 | Fungi;Basidiomycota;Agaricomycetes;Agaricales;Entolomataceae;unc;unc |
| 7 | 2.29 | 79.13;100.00 | Fungi;Basidiomycota;Agaricomycetes;Cantharellales;Cantharellaceae;Craterellus;Craterellus_excelsus |
| 8 | 1.91 | 100.00;100.00 | Fungi;Basidiomycota;Agaricomycetes;Agaricales;unc;unc;unc |
| 9 | 1.69 | 80.77;100.00 | Fungi;---;---;---;---;---;--- |
| 10 | 1.46 | 95.36;98.47 | Fungi;---;---;---;---;---;--- |
| Fla5_s | | | |
| 1 | 35.26 | 92.59;100.00 | Fungi;---;---;---;---;---;--- |
| 2 | 7.91 | 100.00;100.00 | Fungi;---;---;Mortierellales;Mortierellaceae;Mortierella;unc |
| 3 | 6.98 | 80.61;100.00 | Fungi;---;---;---;---;---;--- |
| 4 | 4.07 | 97.74;100.00 | Fungi;---;---;Mortierellales;---;Modicella;Modicella_reniformis |
| 5 | 3.65 | 98.15;100.00 | Fungi;---;---;---;---;---;--- |
| 6 | 3.24 | 99.43;100.00 | Fungi;Ascomycota;Sordariomycetes;Hypocreales;Clavicipitaceae;unc;unc |
| 7 | 1.33 | 89.66;33.73 | Fungi;Chytridiomycota;Chytridiomycetes;Chytridiales;Chytriomycetaceae;Chytriomyces;unc |
| 8 | 1.26 | 95.36;98.47 | Fungi;---;---;---;---;---;--- |
| 9 | 1.25 | 82.42;100.00 | Fungi;---;---;---;---;---;--- |
| 10 | 1.18 | 79.13;100.00 | Fungi;Basidiomycota;Agaricomycetes;Cantharellales;Cantharellaceae;Craterellus;Craterellus_excelsus |

**Supplementary Figures**

| (a)  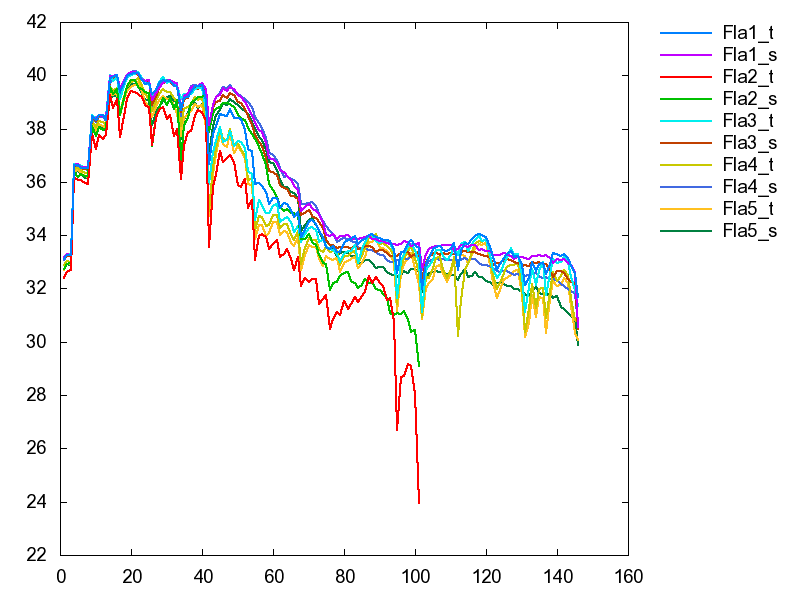 | (b)  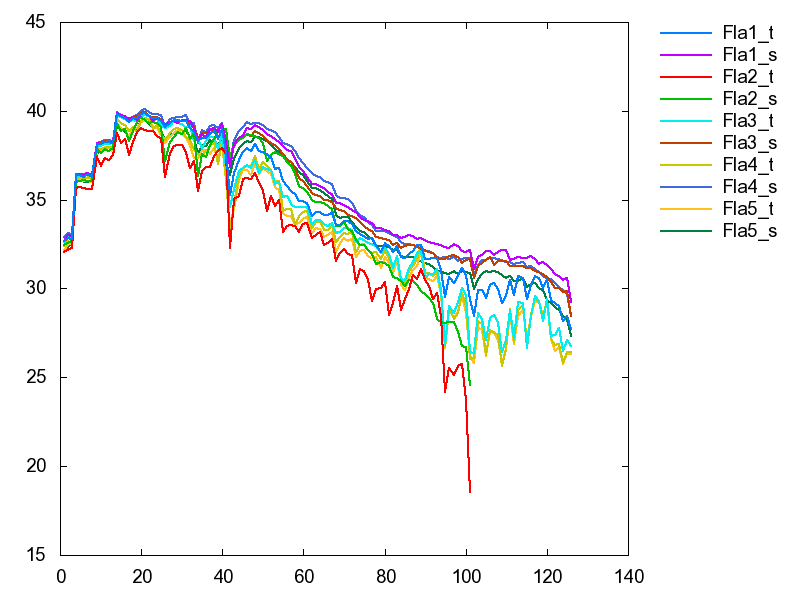 |
| --- | --- |

Figure S1. Mean quality at each base position of (a) read1 and (b) read2 of *Gastrodia flavilabella* (Fla) tuber (t) and the surrounding soil (s) samples.


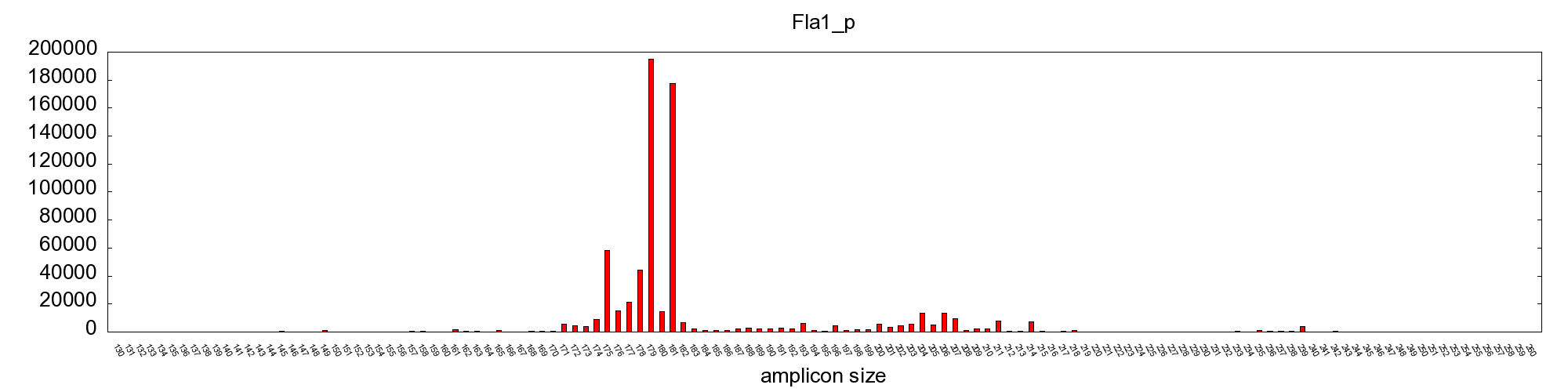

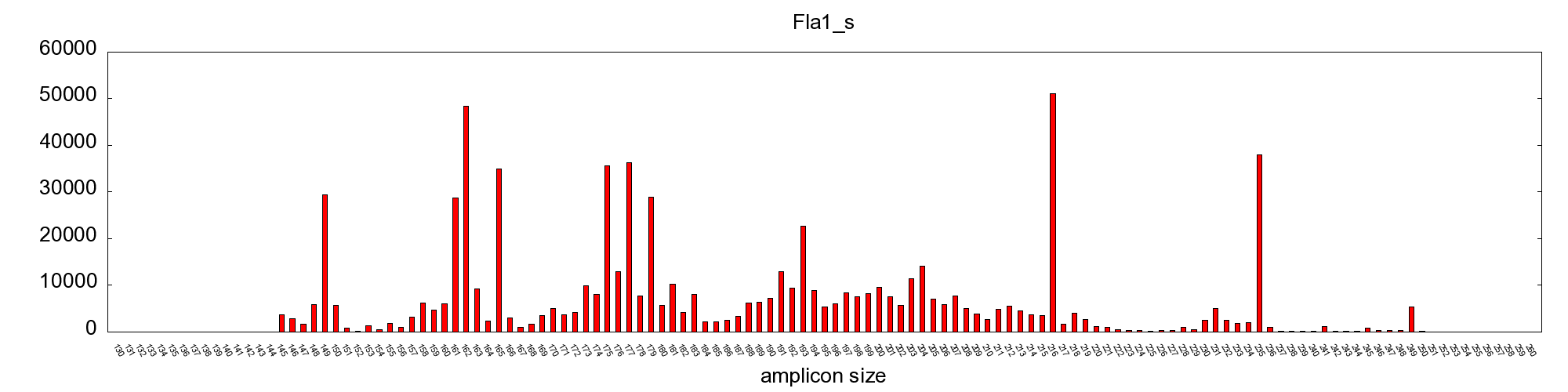

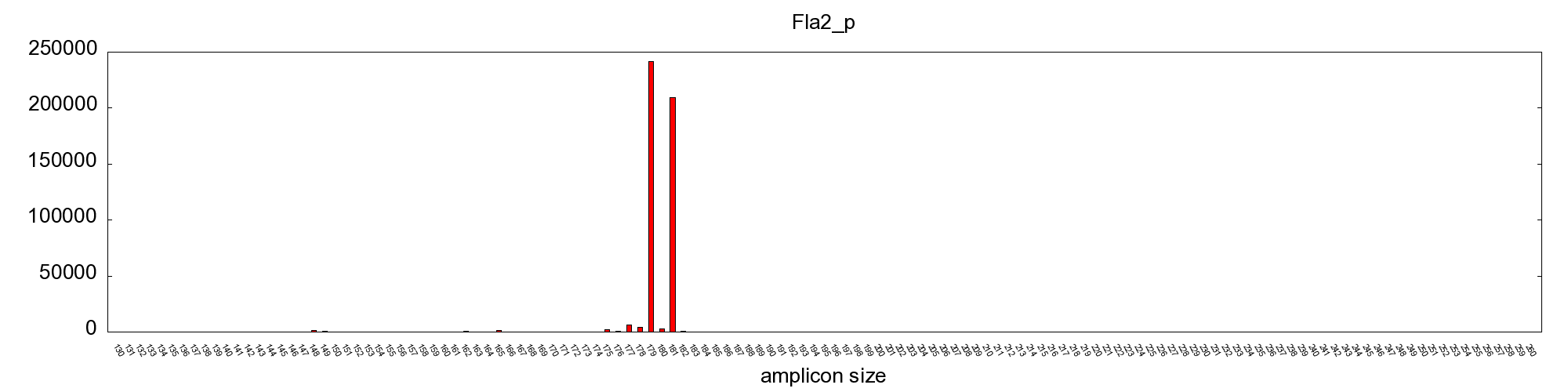

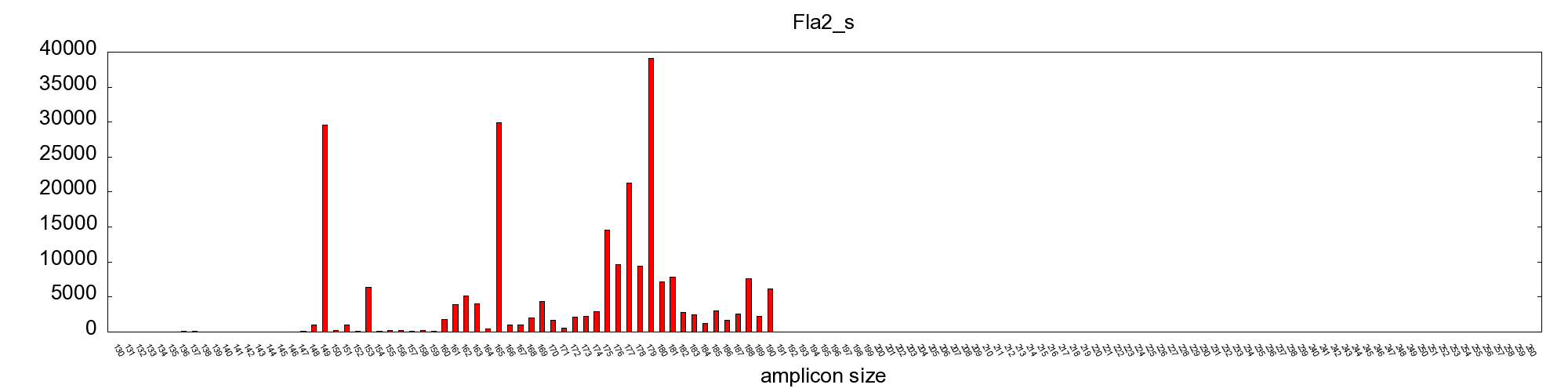

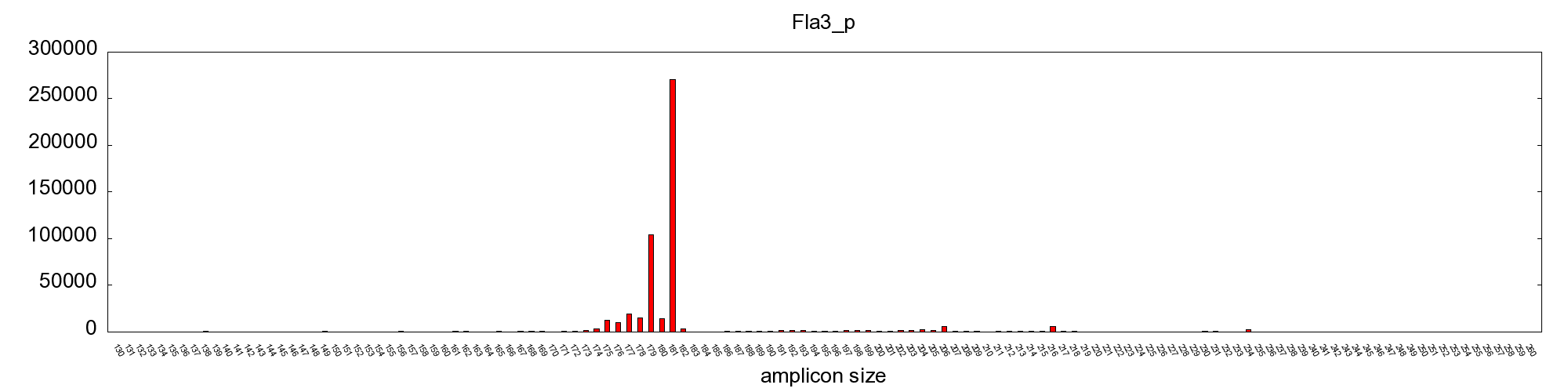

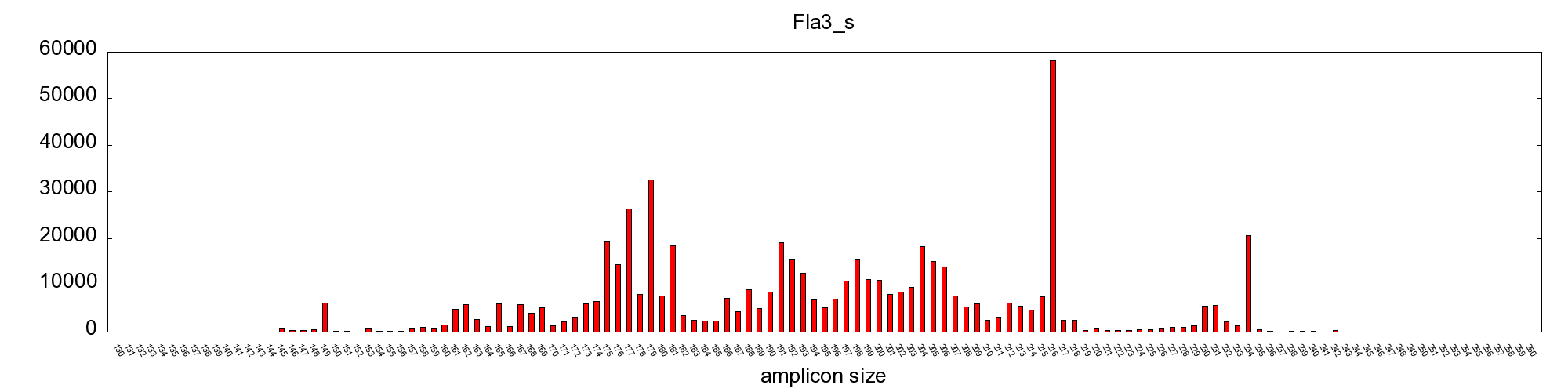

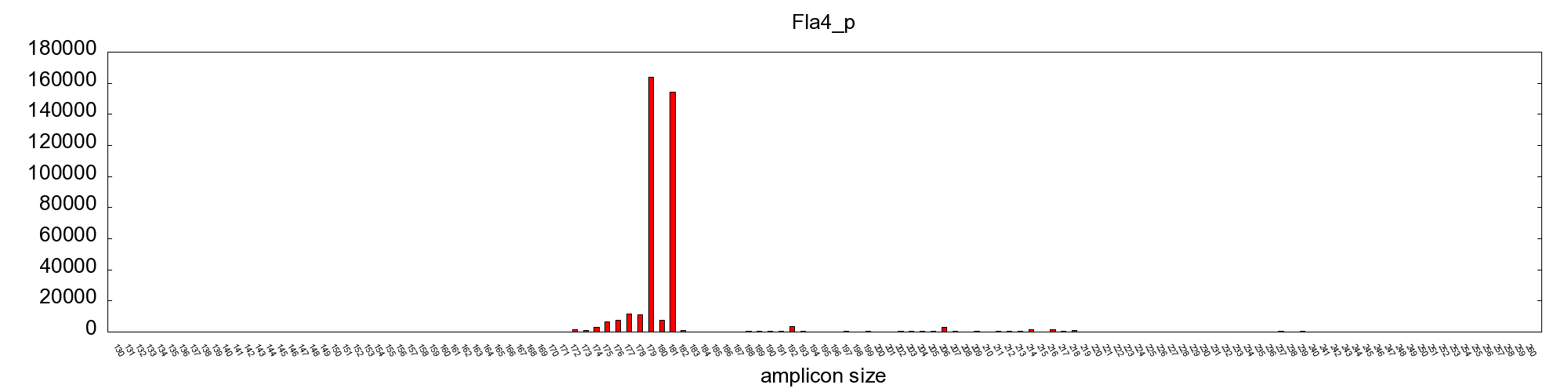

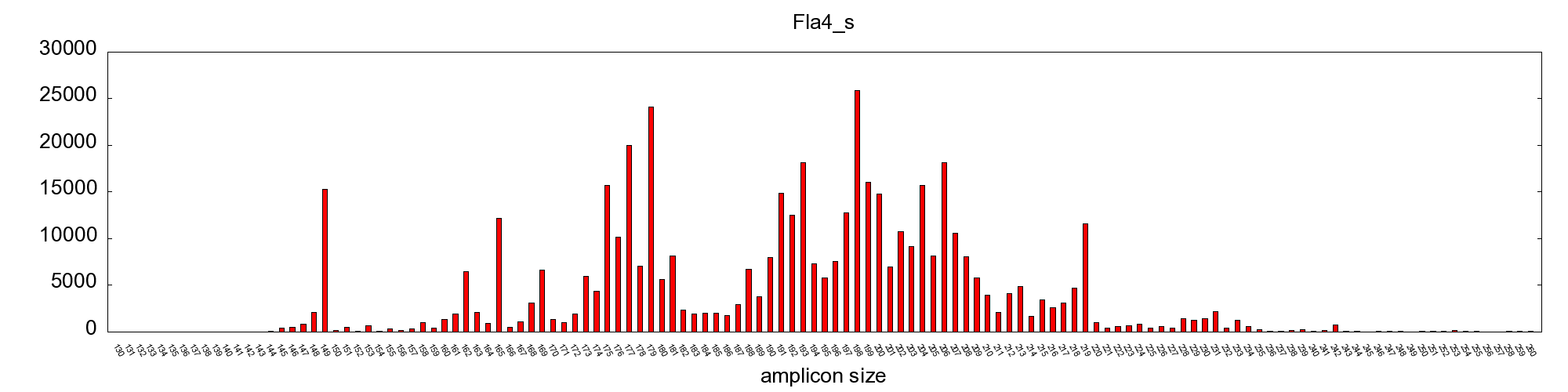

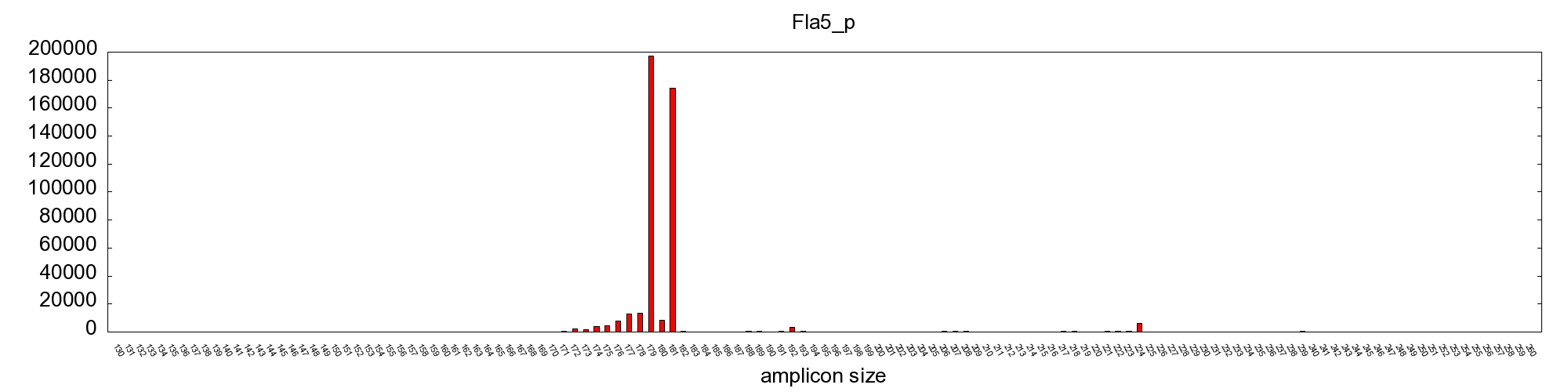

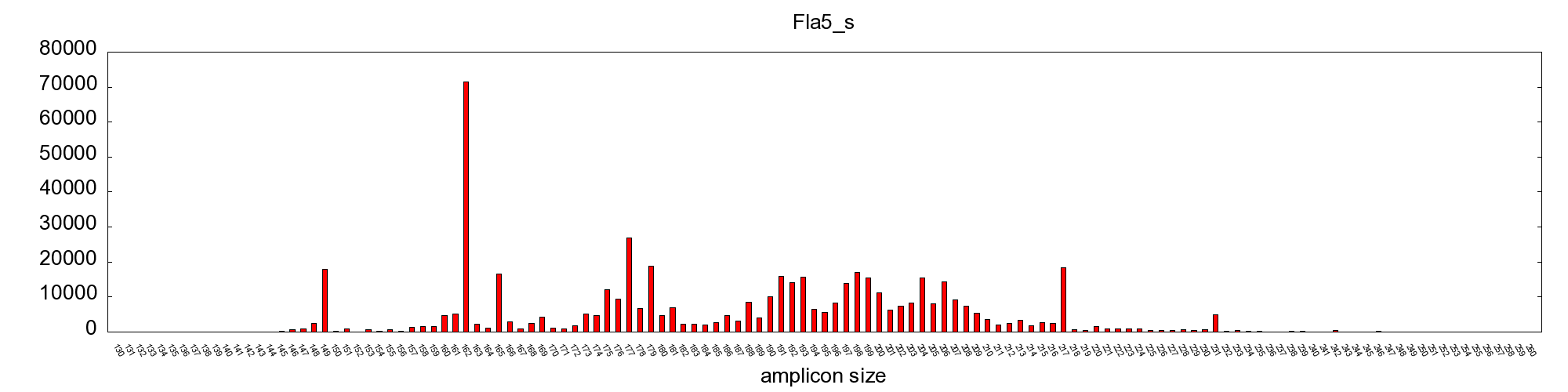

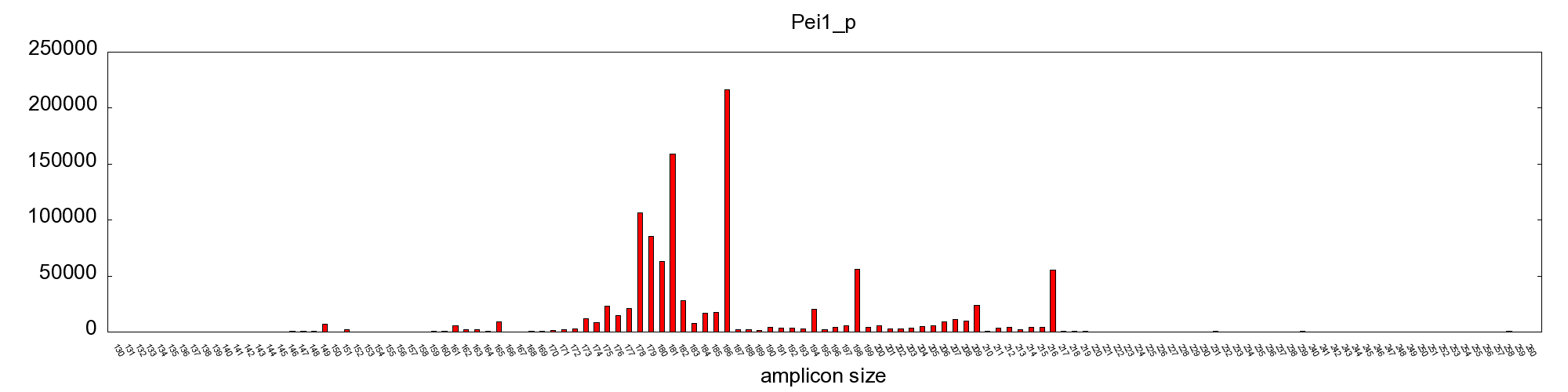

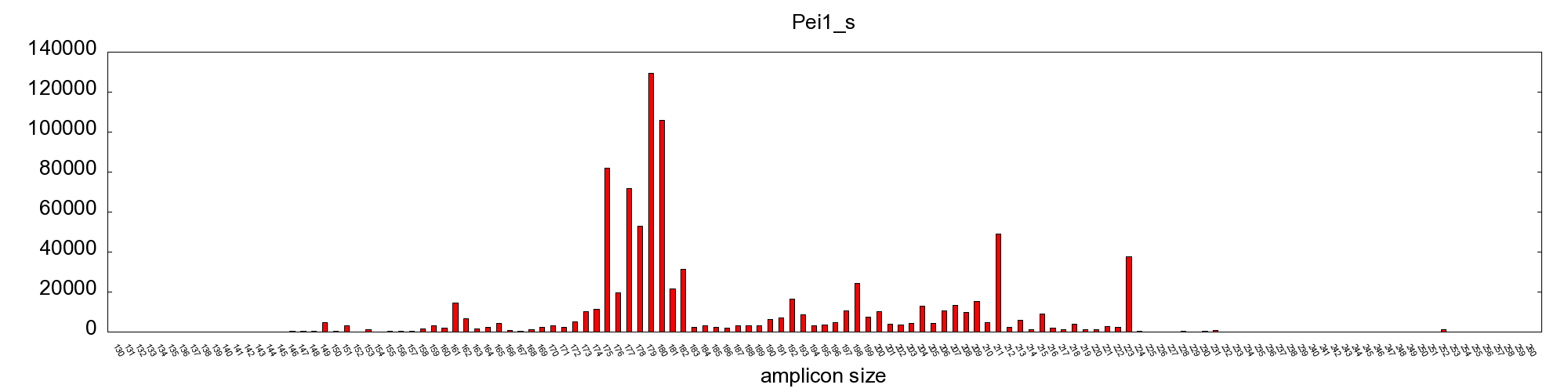


Figure S2. Length distribution of the merged reads, i.e., amplicons, of the *Gastrodia flavilabella* tuber and soil samples.

| Fla1 tuber  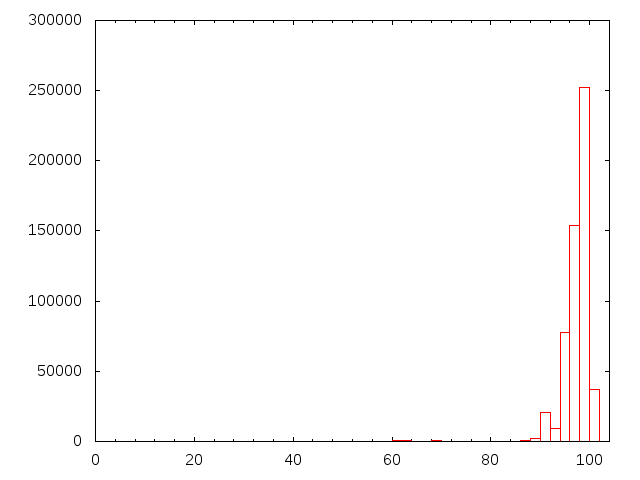 | Fla1 soil  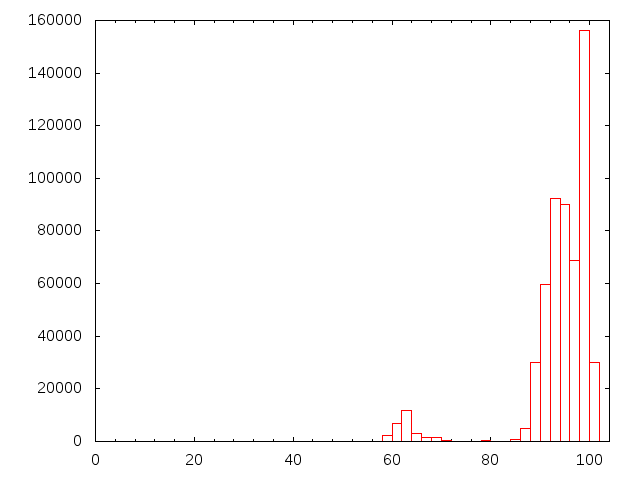 |
| --- | --- |
| Fla2 tuber  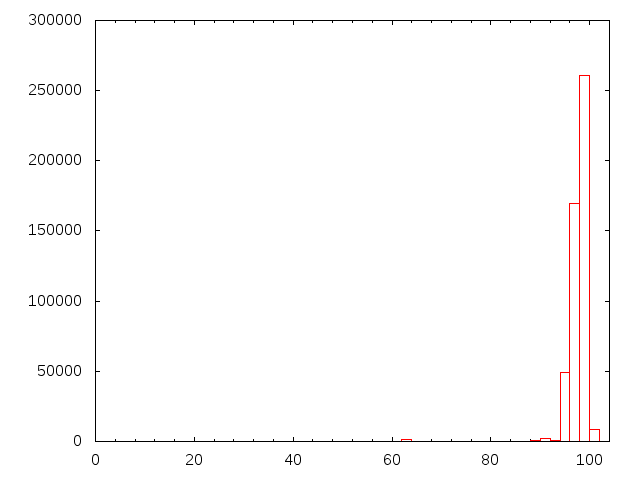 | Fla2 soil  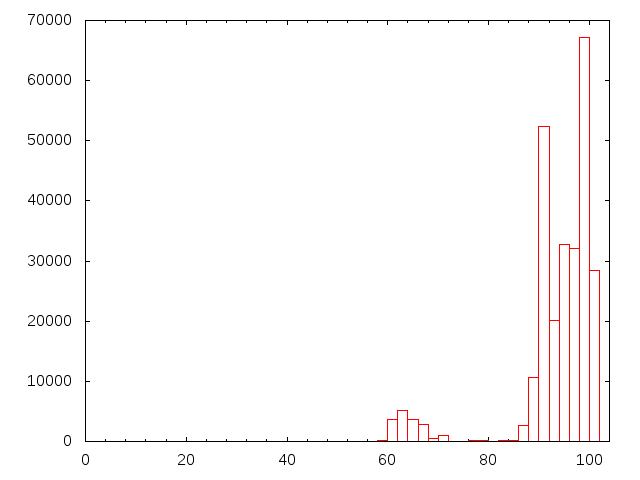 |
| Fla3 tuber  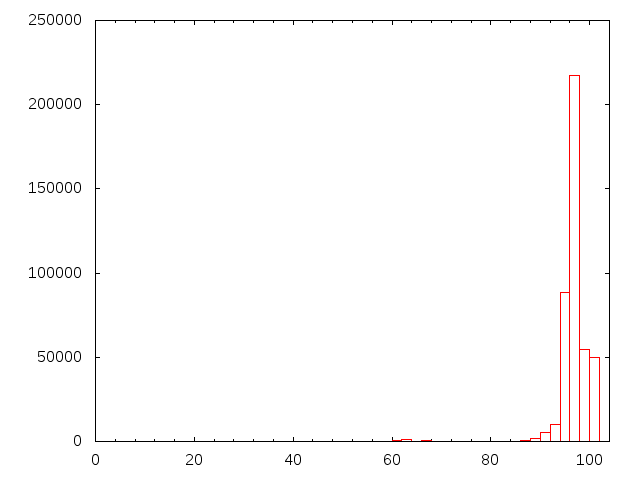 | Fla3 soil  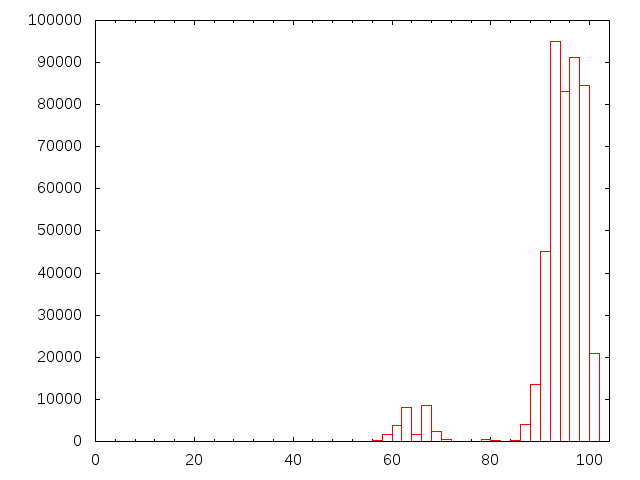 |
| Fla4 tuber  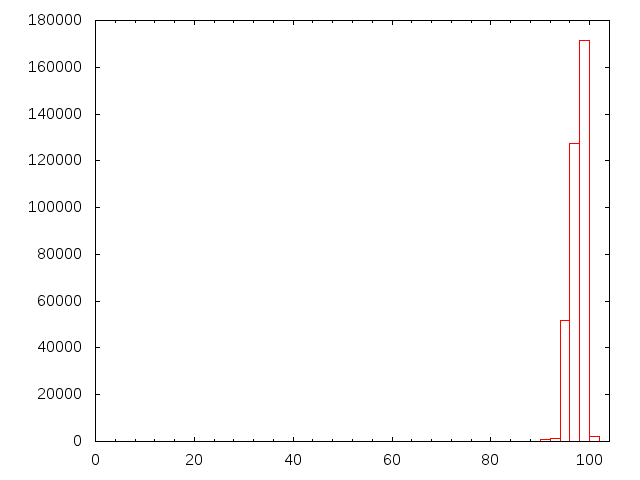 | Fla4 soil  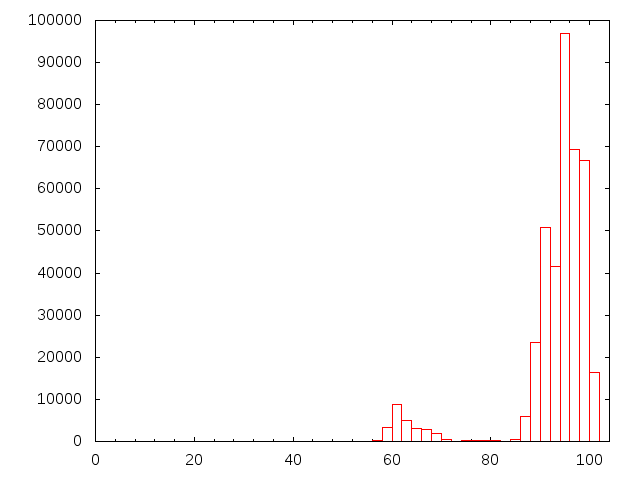 |
| Fla5 tuber  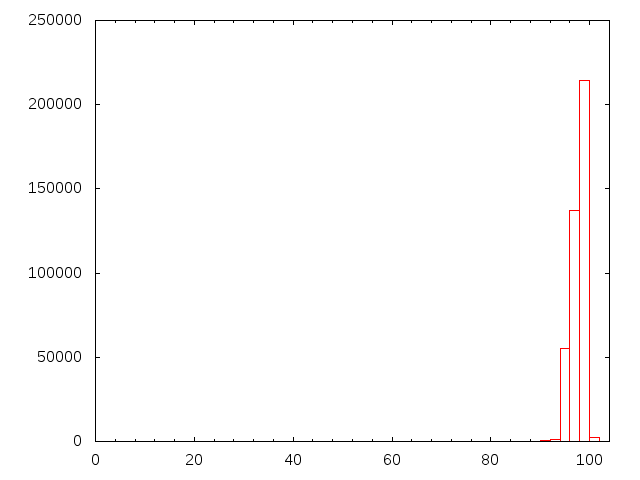 | Fla5 soil  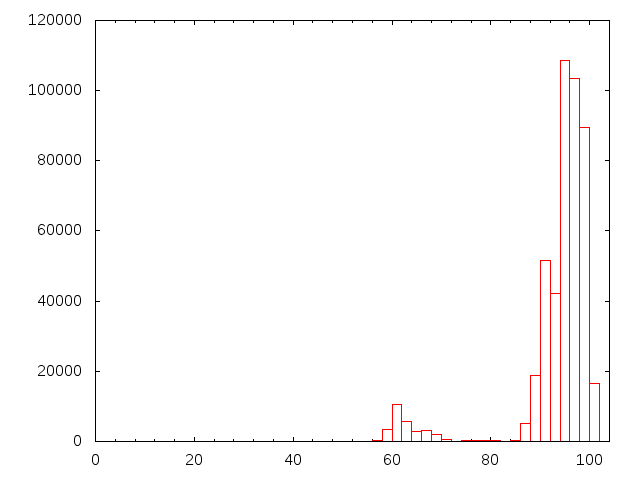 |

Figure S3. Distributions of alignment identities of the merged reads to NCBI LSU reference sequences for *Gastrodia flavilabella* tuber samples and the surrounding soil samples.

(a)

| Phylum  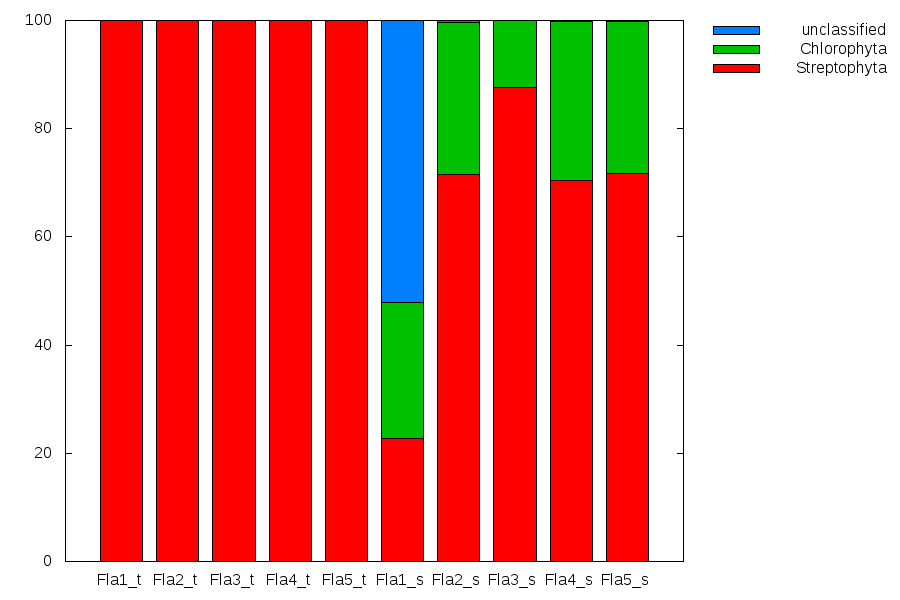 | Class  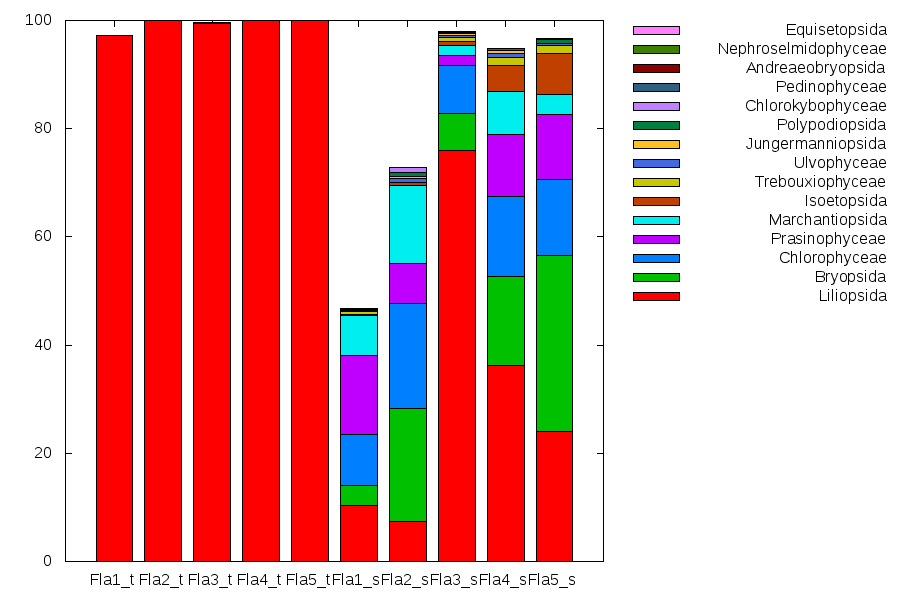 |
| --- | --- |
| Order  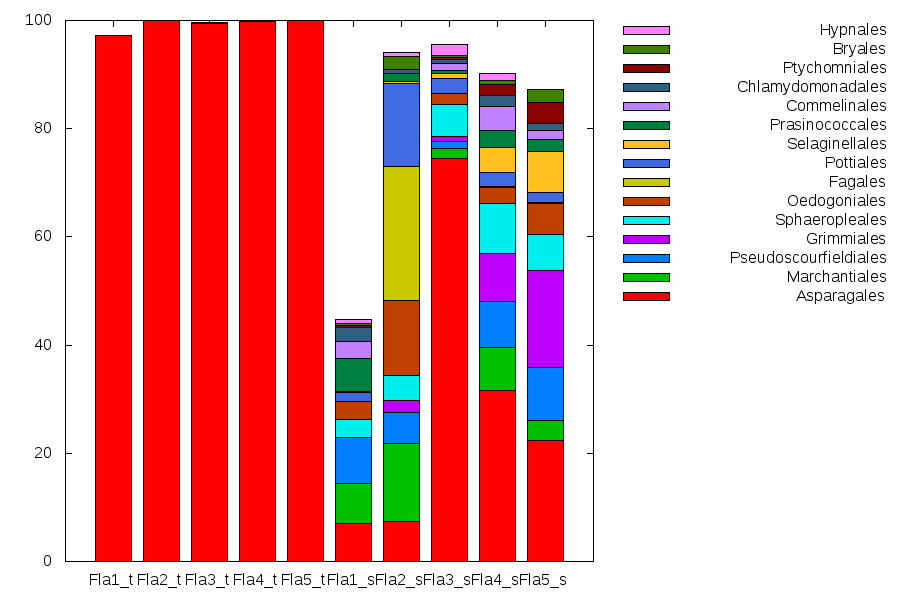 | Family  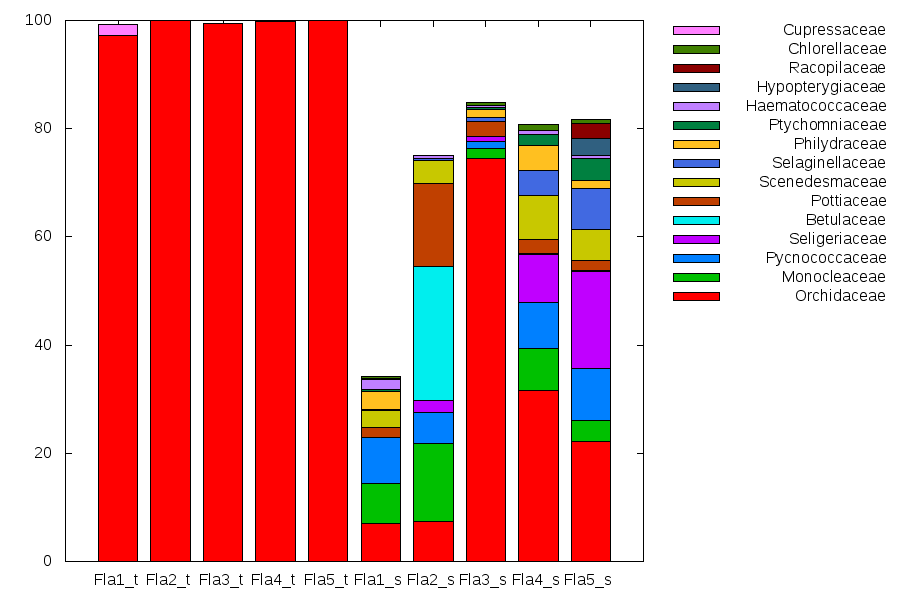 |
| Genus  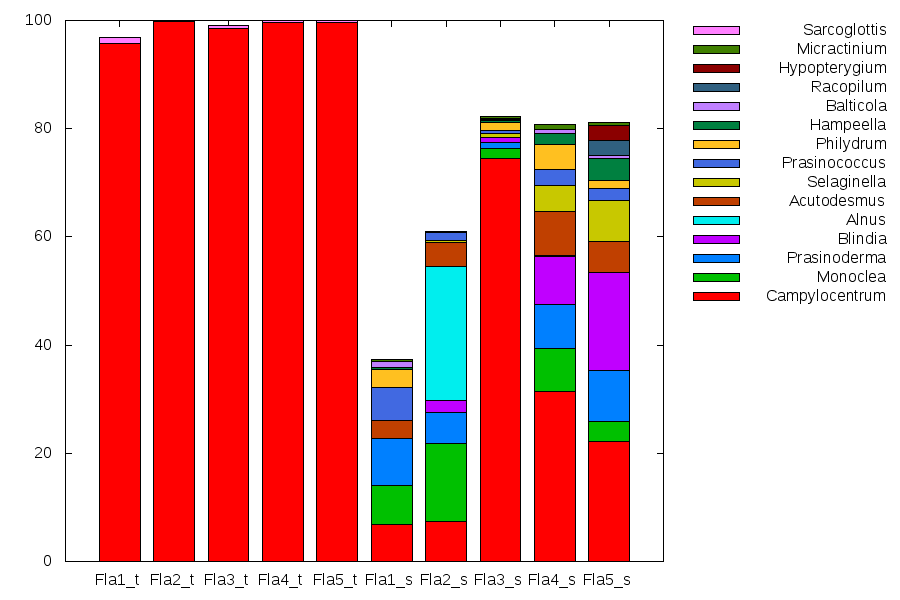 | Species  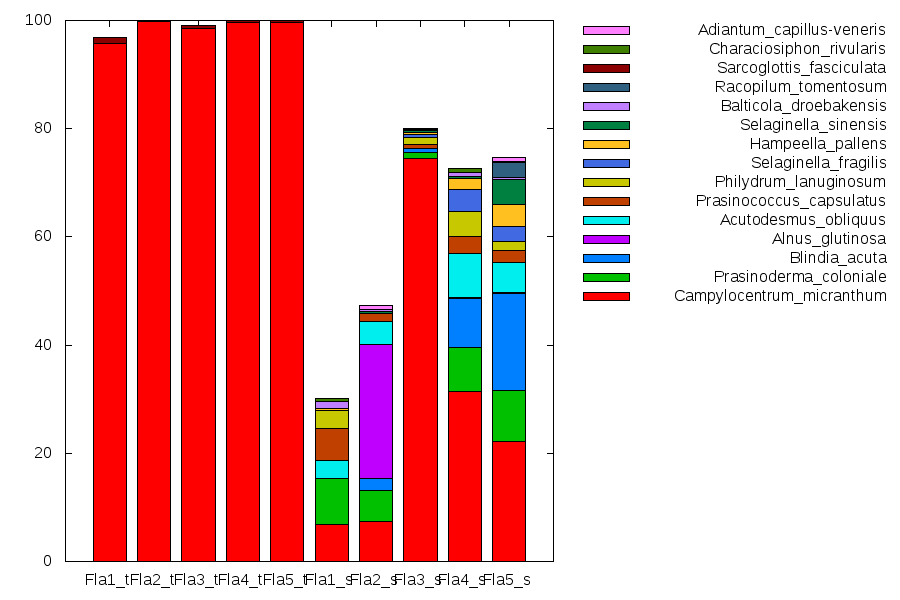 |

(b)

| Phylum  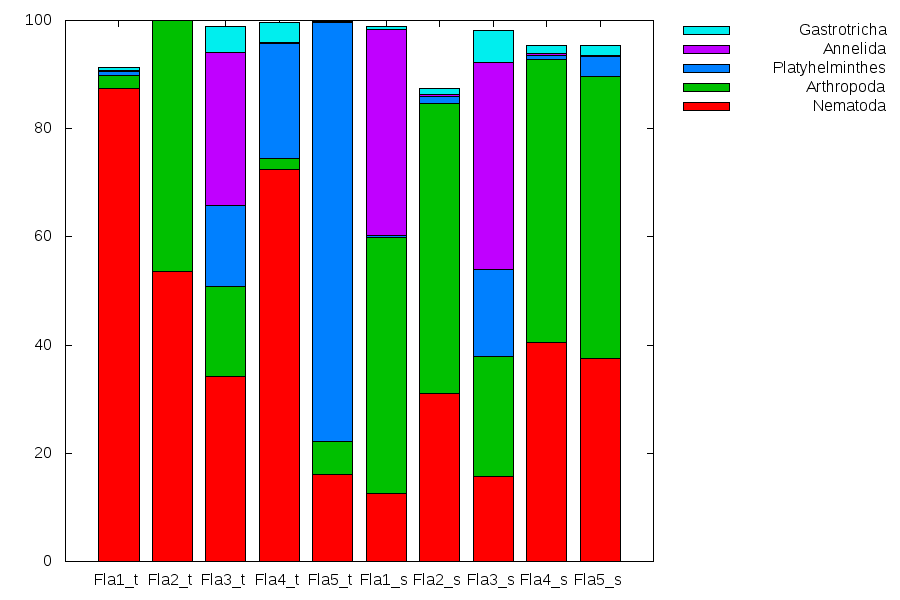 | Class  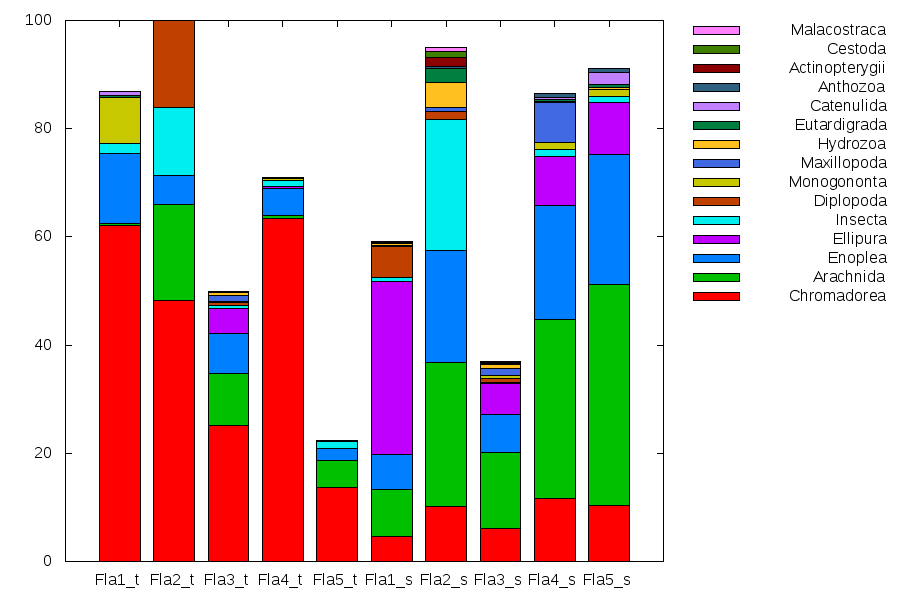 |
| --- | --- |
| Order  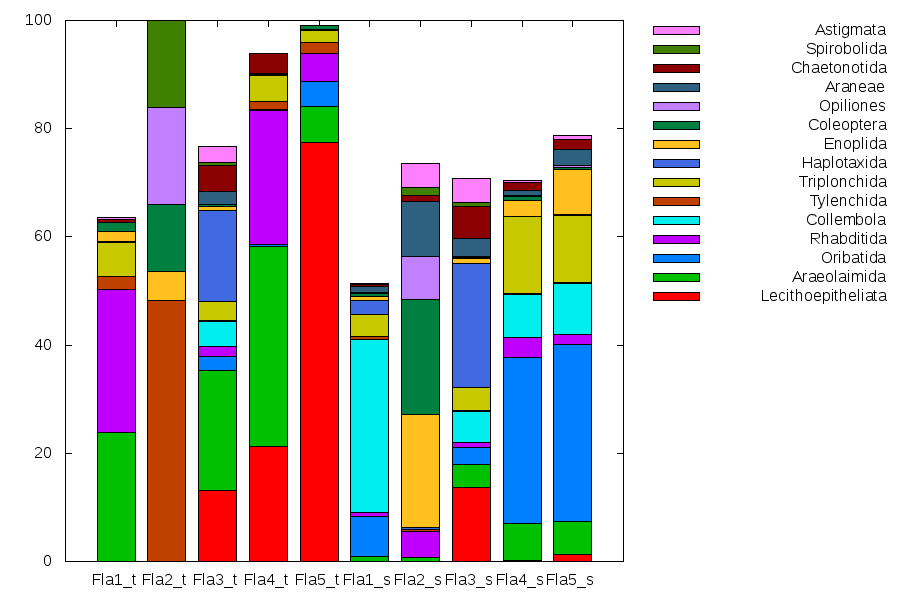 | Family  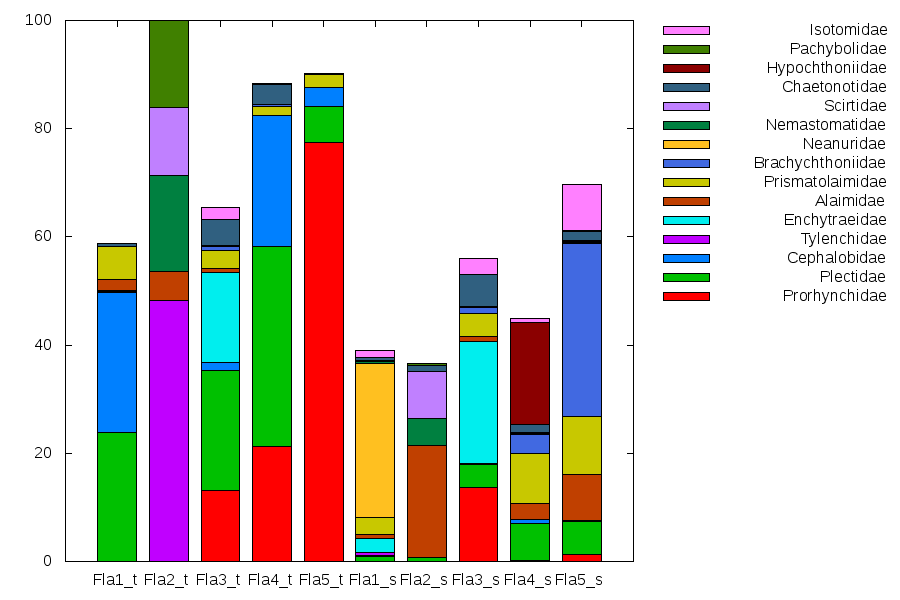 |
| Genus  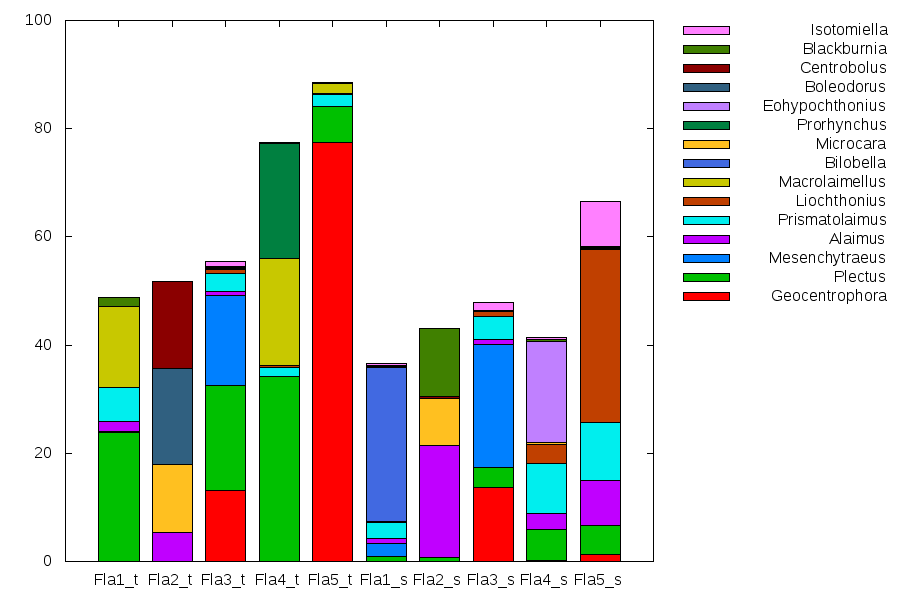 | Species  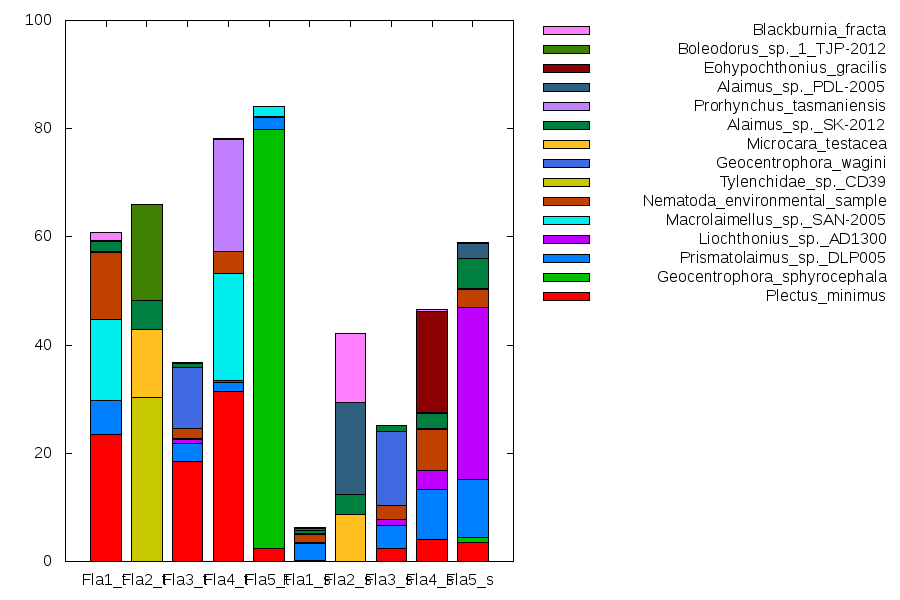 |

Figure S4. Compositions of (a) viridiplantae and (b) metazoan taxonomies.

| (a)  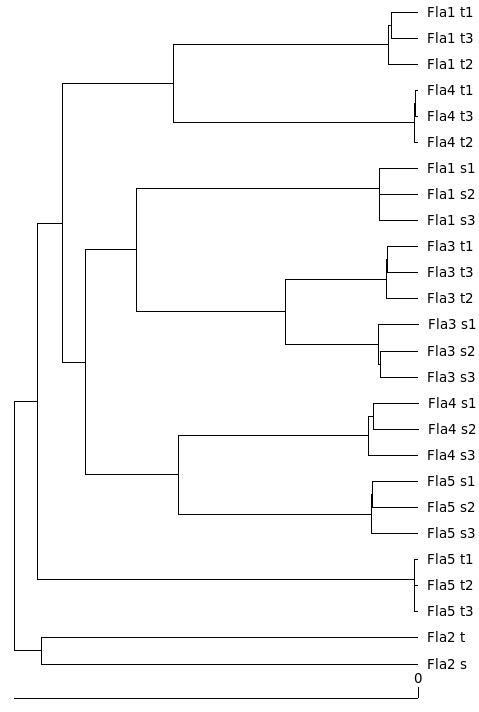 | (b)  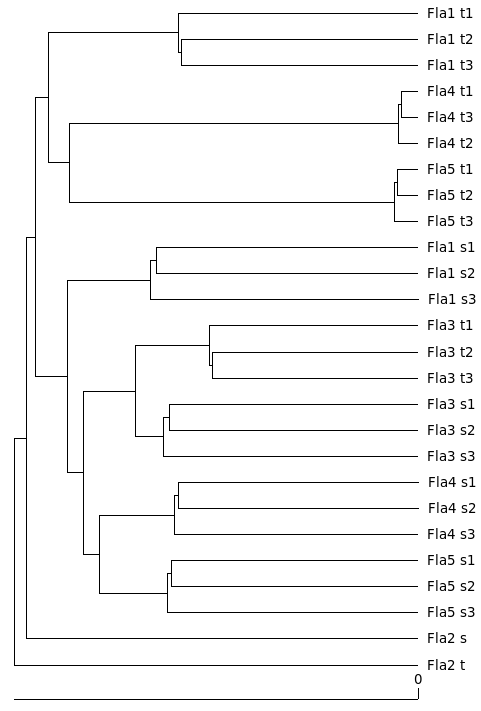 |
| --- | --- |

Figure S5. Clustering of metazoan communities in *Gastrodia flavilabella* tubers and the surrounding soil: (a) weighted and (b) unweighted UniFrac analysis.
